# Stability of grain and fodder yields in extra-early cowpea genotypes in Southeastern Niger

**DOI:** 10.1371/journal.pone.0354617

**Published:** 2026-07-28

**Authors:** Souleymane Abdou, Abdoul Moumouni Iro Sodo

**Affiliations:** 1 Institut National de la Recherche Agronomique du Niger (INRAN), Niamey, Niger; 2 Institut National de la Recherche Agronomique du Niger (INRAN), Maradi, Niger; University of Agriculture Faisalabad, PAKISTAN

## Abstract

Cowpea [*Vigna unguiculata* (L.) Walp.] is a vital food and fodder crop in Niger, but its productivity is constrained by drought, poor soils, striga and pest pressures. Developing extra-early dual-purpose varieties is essential for strengthening food and feed security in these fragile production zones. This study evaluated the effects of genotype, environment, and their interaction on the stability of grain and fodder yields in 21 cowpea genotypes tested across six environments using an alpha-lattice design with three replications. Stability was assessed through Shukla’s stability variance, the cultivar superiority index, Wricke’s ecovalence, GGE biplot, and the multi-trait genotype–ideotype distance index (MGIDI). Results revealed significant differences among genotypes, environments, and genotype × environment interactions for all traits; environmental effects had the highest mean squares for all traits (p < 0.001). Broad-sense heritability ranged from 0.77 (fodder yield) to 0.88 (days to 50% flowering), and genetic advance as percentage of mean was high for yield traits (GAM = 78.67% for pod yield, 81.80% for grain yield, 99.59% for fodder yield), indicating strong selection potential. Mean grain yield across all genotypes and environments was 1,394.32 kg ha ⁻ ¹, and the coefficient of variation ranged from 26.59% (GY) to 40.63% (FY). Notably, ten extra-early lines—BM22, BP02, BP15, MM142, 65B5080, BP18, BM21, BM13, MM141, and BM12—were identified as combining early maturity with high and stable grain and fodder yields. These lines should be further evaluated across diverse agroecological zones to confirm their adaptability and alignment with farmer preferences, prior to deployment as released varieties or as parents in cowpea improvement programs targeting climate-resilient, dual-purpose productivity in the Sahel.

## Introduction

Cowpea is a warm-season annual legume that plays a vital role in food security due to its high nutritional and economic value. Its ability to tolerate drought and low-fertility soils, together with its multiple uses as food, animal feed, and a source of income, has made it a key legume crop in Sub-Saharan Africa [1–3]. In Niger as in other SSA countries, cowpea is consumed in diverse forms—such as boiling in mixtures with rice, soups (gnabbe), or processed into cowpea pancake (*kossai)* and *dan wake* among others. Beyond human consumption, cowpea haulms and shell are critical livestock feed, particularly for fattening small ruminants.

Niger ranks as the second-largest producer of cowpea, with an estimated annual production of about 2.6 million metric tons, following Nigeria at approximately 3.6 million metric tons [4]. Cowpea is well adapted to harsh environments due to its high tolerance to drought and capacity for biological nitrogen fixation, making it particularly suitable for hot, water-limited agro-ecologies zones [5,6]. This resilience is especially important for smallholder farming systems, where cowpea serves as a key source of household income and dietary protein [7,8]. Production remains constrained by multiple biotic and abiotic stresses, including drought, low soil fertility, *Striga* infestation, insect pests, and diseases, compounded by the predominance of unimproved varieties on smallholder farms [9]. Climate change further aggravates these constraints through erratic rainfall, prolonged droughts, and elevated pest pressure. In addition, Low productivity on smallholder farms is further exacerbated by the widespread cultivation of unimproved local varieties and the use of suboptimal crop management practices. Given these constraints, region-specific breeding strategies are therefore required to improve cowpea performance under shifting climatic conditions [10,11]. In areas characterized by terminal drought and high late-season insect pressure, short-duration cowpea varieties capable of producing high grain and/or fodder yields represent a practical and effective option for farmers. Consequently, breeding programs have increasingly emphasized the development of genotypes that combine high productivity with tolerance to both abiotic and biotic stresses [5,12]. However, genotype × environment interactions play a major role in cowpea performance, making the reliable identification of high-yielding and stable varieties across environments challenging [13–17].

While some genotypes maintain stable performance across diverse conditions, others exhibit adaptation to specific environments, indicating the importance of multi-environment trials for determining whether genotypes exhibit broad or narrow adaptation [15,18,19]. At the same time, modern cowpea improvement efforts require the simultaneous enhancement of multiple traits, a task made challenging by unfavorable trait correlations and complex genetic control [20–22]. To address these challenges, a wide range of statistical methodologies has been developed, ranging from traditional analysis-of-variance approaches to more advanced univariate and multivariate parametric methods. Conventional breeding strategies based on single-trait selection are often inadequate when genetic variation is limited or when negative correlations exist among traits, and they become inefficient when multiple traits must be improved concurrently. By contrast, multi-trait selection methods that combine mixed-model analysis with restricted maximum likelihood (REML) and best linear unbiased prediction (BLUP) provide a more robust and reliable basis for selection decisions [23,24]. To support multi-trait selection, several indices such as the Smith–Hazel (SH) index, the factor analysis and ideotype design (FAI-BLUP) index, the multi-trait genotype–ideotype distance index (MGIDI) have been developed [20,24–26]. In the context of high terminal drought incidence and intense late season insects’ pressure, short duration varieties with high grain and/or fodder yields are most appropriate alternatives in improving cowpea production on farmers’ fields. The use of high yielding early improved varieties was recommended among the adaptation measures to variability and climate change in Niger [9,27]. Among the extra-early cowpea genotypes developed at INRAN, it is possible to identify lines that combine high grain and fodder yield with stability across contrasting environments without compromising the phenological advantage of extra-early maturity—a key adaptive trait in Niger’s short and erratic rainy seasons. The objective of this study was to identify extra-early maturing cowpea lines that combine high grain and fodder yields with stability across multiple environments.

## Materials and methods

### Genetic material

Twenty-one extra-early cowpea genotypes were evaluated, comprising 19 newly developed breeding lines and two standard check varieties (UAM09_1055_6 and IT99K573_1_1) (Table 1). The breeding lines were developed at the Institut National de la Recherche Agronomique du Niger (INRAN) using the bulk selection approach. Among these lines, fifteen originated from the cross N’Diambour × IT84S-2246–6, three from G118 × IT87D-1083, and one from G150 × IT87D-1083. The parental lines N’Diambour, G118, and G150 are characterized by high grain and fodder yields under low-phosphorus conditions, whereas IT84S-2246–6 and IT87D-1083 are early-maturing but comparatively low-yielding.

**Table 1:** List of the evaluated genotypes. Test (T), check (C).

Genotypes	Pedigree	Seed colour	Seed size	Status
ML23	N’Diambour x IT84S-2246–6	Brown	Medium	T
ML04	N’Diambour x IT84S-2246–6	Brown	Medium	T
65B 5080	N’Diambour x IT84S-2246–6	White	small	T
BM22	N’Diambour x IT84S-2246–6	White	Small	T
BM23	N’Diambour x IT84S-2246–6	White	Small	T
BM12	N’Diambour x IT84S-2246–6	White	Medium	T
MM98	G118 x IT87D-1083	Brown	Medium	T
MM142	N’Diambour x IT84S-2246–6	Brown	Small	T
ML26	N’Diambour x IT84S-2246–6	Brown	Medium	T
MM156	N’Diambour x IT84S-2246–6	Brown	Small	T
BM13	N’Diambour x IT84S-2246–6	White	Medium	T
BP15	N’Diambour x IT84S-2246–6	White	Medium	T
BL22	G150 x IT87D-1083	White	Medium	T
BL18	G118 x IT87D-1083	White	Large	T
MM141	N’Diambour x IT84S-2246–6	Brown	Small	T
BP18	N’Diambour x IT84S-2246–6	White	Small	T
BM21	N’Diambour x IT84S-2246–6	White	Medium	T
MM65	G118 x IT87D-1083	Brown	Medium	T
BP02	N’Diambour x IT84S-2246–6	White	Small	T
UAM09_1055_6		White	Large	C
IT99K573_1_1		White	Large	C

### Experimental sites, layout and data collection

Field experiments were carried out during the 2021 and 2022 rainy seasons at three locations in southern Niger: Konni (13°82′ N, 5°28′ E), Maradi (15°26′ N, 8°33′ E), and Magaria (12°59′ N, 6°56′ E). The three sites were selected to represent the main agro-climatic zones of southern Niger where cowpea is commercially produced. The soils across the experimental sites were predominantly sandy, although some variation in texture was observed. Maradi soil consisted of 72% sand, 11% silt, and 17% clay, while Magaria soil was more sandy, with 88% sand, 4% silt, and 8% clay. whereas Konni soils were characterized by 70% sand, 10.2–30% silt, and 15–20% clay. Soil organic carbon content was generally low across all sites, indicating poor soil fertility. Values were 0.30% at Maradi, 0.27% at Magaria, and 0.15% at Konni. Soil pH ranged from slightly acidic to near neutral, with values of 5.4 at Maradi, 5.8 at Magaria, 5.5 at Konni. Annual rainfall varied across sites and years; however, all the sites received more rainfall in 2022. Total annual rainfall recorded were 323.5, 446.3 and 535.6 mm at Konni, Magaria and Maradi in 2021. In 2022, Konni, Magaria and Maradi received 468.9, 741.39, and 755.2 mm respectively. The trials were laid out in a 3 × 7 alpha-lattice design with three replications. Each genotype was planted in four-row plots, each 4 m long, with 0.5 m spacing between rows and between plants within rows. Fields were ploughed and harrowed prior to sowing. Two to three seeds were placed per hill and thinned to one plant per stand three weeks after emergence. Manual weeding was performed as needed. Insecticide was applied at flowering, pod formation, and pod filling stages to minimize pest damage.

In 2021, no fertilizer was applied, while in 2022, each plant received 6 g of a compound fertilizer (NPK 15:15:15) was used at 6 g per plant three weeks after sowing. Data collected included the number of days to first flowering, days to 50% flowering, days to 50% maturity, and the dried grain and fodder yields (kg/ha).

### Data analysis

Combined analysis of variance (ANOVA) was conducted using the mixed linear model (MLM) implemented in the lme4 package in R software 4.3.1 [28] as follows:


$$\begin{array}{c} \text{Y}=\mu +\text{Rep}+\text{Rep }(\text{Blk})+\text{G}+\text{E}+\text{G}\times \text{E}+\text{e} \end{array}$$

where Y = phenotypic value; µ = grand mean; G = genotypes; E = environment; Rep = replication nested within environment; Rep (Blk) = incomplete blocks nested within replication; G × E = genotype by environment interaction; e = residual.

Phenotypic and genotypic coefficients of variation (PCV and GCV) and genetic advance as a percentage of the mean (GAM) were calculated following the method of Burton and DeVane [29] to estimate trait variability.

The PCV and GCV were calculated and classified into three classes: less than 10% (low), 10–20% (moderate), and more than 20% (high).


$$\begin{array}{c} \text{Phenotypic Coefficient of Variance }(\text{PCV})=\frac{\sqrt{Phenotypevariance}}{\text{Mean}}\text{x100} \end{array}$$

$$\begin{array}{c} \text{Genotypic Coefficient of Variance }(\text{GCV})=\frac{\sqrt{Genotypevariance}}{\text{Mean}}\text{x100} \end{array}$$

$$\begin{array}{c} \text{Heritability }(\text{broad sens})=\frac{\text{Genotype variance}}{\text{Phenotype variance}}\text{X100} \end{array}$$

$$\begin{array}{c} \text{Genetic advance }(\text{GAM}\%)=\frac{\text{K }*\text{ H }*\text{ p }}{\text{Mean }}\text{x100} \end{array}$$

where: K = 2.06 at 5% selection intensity; H = Heritability; P = Phenotypic standard deviation. The GAM was categorized into three classes: < 10% (low), 10–20% (moderate), and >20% (high) [30].

To assess the stability of genotype performance across environments, several stability metrics were calculated, including Shukla’s stability variance [31], the cultivar superiority index [32], Wricke’s ecovalence [33], and the GGE biplot analysis. The GGE biplot methodology, derived from graphical analysis of multi-environment trials, was used to visualize genotype-by-environment interactions and identify genotypes with broad or specific adaptability. Pearson correlation coefficients for the evaluated traits were assessed using R [28]. These stability statistics were selected for their complementary strengths: Shukla’s stability variance and Wricke’s ecovalence both quantify static (biological) stability by measuring variance in genotype performance across environments; the cultivar superiority index (Pi) simultaneously integrates yield level and stability by penalizing deviation from the highest-yielding genotype per environment; and the GGE biplot provides visual diagnostic capability for identifying mega-environments and comparing genotypes graphically. Shukla’s stability variance was determined by estimating the deviation of each genotype’s observed performance from its expected performance across environments, standardized relative to the overall mean across all genotypes and environments.


$$\begin{array}{c} Si=\text{1}-\frac{\left(\text{Yi}.-\overset{―}{\text{Y}}\right)}{\left(\text{Yij }-\overset{―}{\text{ Y}}\right)} \end{array}$$

where Si represents the stability of genotype i; Yi is the mean performance of genotype i across all environments; Ῡ is the overall mean of all genotypes and environments; Yij = performance of genotype i in environment j.

The cultivar superiority index (Pi) was computed as the average squared deviation between a genotype’s trait value and the maximum observed value in each environment, averaged across all environments:


$$\begin{array}{c} Pi=\frac{\text{1}}{\text{2}n}\sum_{j=i}^{n}(\text{Xij }-\text{ Mj}{)}^{\text{2}} \end{array}$$

where P_*i*_ denotes the superiority index of the *i-th* genotype; X_ij_ is the trait value of i-th genotype in the *j-th* environment; M_*j*_ is the maximum trait value from all the genotypes in the *j-th* environment; and n is the total number of environments. Wricke’s ecovalence (Wi) was applied to measure the extent to which each genotype contributes to the overall genotype × environment interaction. Genotypes with lower Wi values are considered more stable, as their performance is less affected by environmental factors.


$$\begin{array}{c} {W_{i}=\left(Y_{ij}-Y_{i}-Y_{j}-Y\right)}^{2} \end{array}$$

where *W*_i_ denotes ecovalence of the *i-th* cultivar;

Y_ij_ denotes the reported trait value of the *i-th* genotype in the *j-th* environment;

*Yi* denotes mean of *i-th* cultivar across environments;

*Y.j* denotes mean of *j-th* environment;

*Y.* denotes grand mean.

The MGIDI proposed by Olivoto [20] was used to select genotypes that combine high grain and fodder yields and earliness using 50% selection intensity. The Multiple-Trait Genotype-Ideotype Distance Index (MGIDI) is based on four key principles: (i) rescaling trait values to a range of 0–100, (ii) considering the correlation structure among traits and reducing the data’ dimensionality, (iii) using desired trait values to plan an ideotype, and (iv) calculating the distance between the planned ideotype and each genotype. The rescaling of trait values was done using the equation provided below:


$$\begin{array}{c} \text{rXij }=\frac{\eta \text{nj }}{\eta \text{oj}}-\frac{\phi \text{nj }}{\phi \text{oj}}*(\Theta \text{ij }-\eta \text{nj})+\eta \text{nj} \end{array}$$

where η_nj_ and ϕ_nj_ represent the new maximum and minimum values for the trait j after rescaling, ϕ_oj_ and ϕ_oj_ represent the original maximum and minimum values for the trait j, and hij represents the original value for the jth trait of the ith genotype. The values for η_nj_ and ϕ_nj_ were chosen as follows. For the traits in which lower gains are desired, then η_nj_ = 0 and ϕ_nj_ = 100 were considered. For the traits where higher gains were desired, η_nj_ = 100 and ϕ_nj_ = 0 were used [34,35]. The ideotype was defined as a hypothetical genotype combining: maximum grain yield (GY), pod yield (PY), and fodder yield (FY)—traits for which higher values are desired (ηnj = 100, ϕnj = 0 in the rescaling formula); and minimum days to first flowering (NDF), days to 50% flowering (ND50F), and days to 50% maturity (ND50MAT)—traits for which lower values are desired (ηnj = 0, ϕnj = 100), consistent with the extra-early maturity breeding objective.

The factorial scores for each genotype were estimated based on the rescaled trait values. To group the correlated traits into factors, a factor analysis was performed according to this following formula:


$$\begin{array}{c} X=\mu +Lf+\epsilon \end{array}$$

Where X (p × 1) represents the rescaled observations, µ (p × 1) is the vector of standardized means, L (p × f) contains the factor loadings, f (p × 1) is the vector of common factors, and ε (p × 1) is the residual vector.

To obtain the initial factor loadings by the traits having more than one eigenvalue that is acquired from the correlation matrix of rXij the final loadings were calculated using varimax rotation criterion as described by [36].


$$\begin{array}{c} F={Z\left(A^{T}R^{-1}\right)}^{T} \end{array}$$

where F represents a g × f matrix with the factorial scores, Z is a g × p matrix with the standardized means (rescaled), A represents a p × f matrix of canonical loadings, R represents a p × p correlation matrix between the traits, and g, f, and p represent the number of evaluated genotypes, factors retained (FA), and traits computed, respectively.

### Ideotype planning

In designing the ideotype (ID), it was considered that the selected genotype has the highest rescaled value (i.e., 100) across all the traits used.


$$\begin{array}{c} \text{MGIDIi}=[\sum_{j=\text{1}}^{f}\left((\gamma \text{ij }-\gamma \text{j}\right)\text{2}{]}^{\text{0}.\text{5}} \end{array}$$

where the MGIDI represents the multi-trait genotype–ideotype distance index for the ith genotype; Yij represents the score of the ith ideotype in the jth factor being g and f; and Yj represents the jth score of the ideotype. From the above formula, the lower the MGIDI score of a genotype, the closer the genotype is to the ideotype. The proportion of the MGIDI of the genotypes explained by the correlated factor is used to show the strengths and weaknesses of the genotypes using the following formula:


$$\begin{array}{c} \omega \text{ij}=\frac{\sqrt{{\text{Dij}}^{\text{2}}}}{\sum_{j=\text{1}}^{f}\sqrt{{\text{Dij}}^{\text{2}}}} \end{array}$$

where ωij is the proportion of the MGIDI of the ith genotype explained by the correlated jth factor, and D2ij is the distance between the ith genotype and the ideotype for the jth factor.

## Results

### Variability in traits

**Fig 1:**
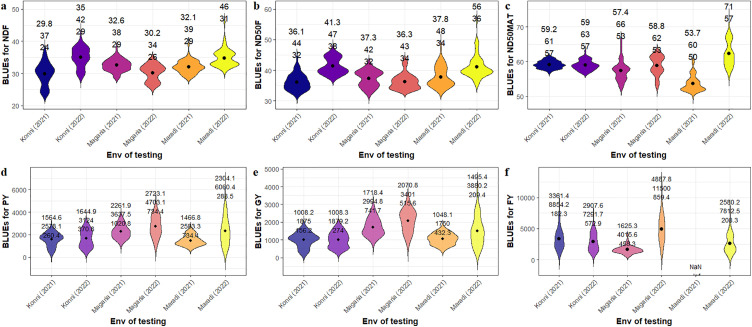
Violin plots showing BLUE distribution of 21 cowpea genotypes evaluated across six environments including Konni (2021 and 2022), Maradi (2021 and 2022), and Magaria (2021 and 2022) for (a) days to first flowering (NDF), (b) number of days to 50% flowering (NDF50F), (c) number of days to 50% maturity (NDF50MAT), (d) pod yield (PY), (e) grain yield (GY), and (f) fodder yield (FY). Black dots represent the mean values, while the vertical spread shows the variability among genotypes. Descriptive statistics (mean, minimum, and maximum) are provided above each plot.

Genotypic performance in each environment was evaluated using best linear unbiased estimates (BLUEs), and the distribution of traits across the various environments is shown in Fig 1. The extent of variability differed markedly among environments. For phenological traits, wider distributions were observed in Konni (2022) and Maradi (2022), indicating stronger environmental effects on time to flowering and maturity. In terms of yield traits, variability was most pronounced in Maradi (2022) and Magaria (2022), suggesting that these environments provided greater discrimination among genotypes. In contrast, Magaria (2021) exhibited both the lowest mean yields and reduced variability, reflecting uniformly poor growing conditions.

Days to first flowering (NDF) ranged from 26 in Magaria (2022) to 46 in Konni (2022), highlighting strong environmental effects on flowering time. A similar trend was observed for days to 50% flowering (ND50F), which varied from 36 in Magaria (2022) to 56 in Konni (2022). Days to 50% maturity (ND50MAT) showed wide variation, from 53 (Magaria 2021 and 2022) to 71 (Maradi 2022), suggesting that maturity duration was highly influenced by season and environmental differences. Magaria (2022) appeared most favorable for early maturity, while Konni (2022) and Maradi (2022) showed delayed reproductive development. A significant effect of the environment was reported for the yield-related traits. Pod yield (PY) ranged from 604 kg ha ⁻ ¹ at Magaria (2021) to 2,294 kg ha ⁻ ¹ at Maradi (2022). Grain yield (GY) was highest at Maradi (2022) (1,495 kg ha ⁻ ¹), followed by Magaria (2022) (1,208 kg ha ⁻ ¹), and lowest at Magaria (2021) (774 kg ha ⁻ ¹). Fodder yield (FY) varied considerably, with Magaria (2022) producing the highest mean (4,887 kg ha ⁻ ¹) and Magaria (2021) the lowest (1,625 kg ha ⁻ ¹).

Overall, Maradi (2022) was the most productive environment for pod and grain yield, while Magaria (2022) favored fodder production. In contrast, Magaria (2021) consistently produced the lowest performance across the yield traits, reflecting a less favorable growing condition.

Results from the combined ANOVA indicated that environmental variation, genotypic differences, and genotype × environment interactions had highly significant effects (p < 0.001) on all evaluated traits (Table 2). Among these sources of variation, the environment contributed the largest mean squares across traits, indicating that environmental conditions exerted a strong influence on trait expression. Mean values were 32.42 days for NDF, 38.32 days for ND50F, and 58.39 days for ND50MAT, with relatively low coefficients of variation (CV%) ranging from 2.61 to 6.19%, indicating good experimental precision. In contrast, yield traits showed greater variability, with mean values of 1,995.16 kg ha ⁻ ¹ for PY, 1,394.32 kg ha ⁻ ¹ for GY, and 3,072.40 kg ha ⁻ ¹ for FY, and high CVs ranging from 26.59 to 40.63%, suggesting a stronger environmental influence on productivity-related traits. Analysis of variance components indicated that phenotypic variance (δ²p) was consistently greater than genotypic variance (δ²g) for all traits, highlighting the contribution of environmental effects to trait expression. Nevertheless, broad-sense heritability estimates were high, ranging from 0.77 for fodder yield to 0.88 for days to 50% flowering, indicating substantial genetic control. The genotypic and phenotypic coefficients of variation were moderate to high for yield traits, whereas they were comparatively low for phenological traits. The narrow differences observed between GCV and PCV for NDF, ND50F, and ND50MAT further suggest limited environmental influence and strong genetic control of these traits. In contrast, larger GCV–PCV differences for yield related traits imply substantial environmental effects. Genetic advance expressed as a percentage of the mean (GAM) was high for all yield-related traits, with values of 78.67% for pod yield (PY), 81.80% for grain yield (GY), and 99.59% for fodder yield (FY).

**Table 2:** Combined analysis of variance (ANOVA), variance components, and genetic variability estimates for all evaluated traits. Environment (Env), genotype (Geno), genotype × environment interaction (Geno × Env), coefficient of variation (CV), phenotypic variance (δ²p), genotypic variance (δ²g), environmental variance (δ²e), phenotypic coefficient of variation (PCV, %), genotypic coefficient of variation (GCV, %), broad-sense heritability (H²), genetic advance as a percentage of the mean (GAM), days to first flowering (NDF), days to 50% flowering (NDF50F), days to 50% maturity (NDF50MAT), pod yield (PY), grain yield (GY), and fodder yield (FY).

Source of variation	DF	NDF	ND50F	ND50MAT	PY	GY	FY
Env	5	306.35***	340.5***	492.94***	133693***	273194.4***	448022***
Geno	20	40.90***	54.03***	42.11***	60999.9***	24955.5***	60743***
Geno: Env		6.22***	7.61***	6.87***	3109.4***	7651.6***	76222***
Residuals		4.02	4.25	2.33	6725.50	7478.30	58651.00
CV (%)		6.19	5.38	2.61	29.94	26.59	40.63
Min		24	32	50	260.42	156.25	182.29
Max		46	56	71	6060.42	3880.21	11500
Mean		32.42	38.32	58.39	1995.16	1394.32	3072.4
δ^2^p		6.03	6.99	4.36	487007.50	208642.47	2019809.10
δ^2^g		2.08	2.76	2.01	126840.90	68594.77	436318.10
δ^2^e		3.95	4.23	2.36	360166.60	140047.70	1583491.00
PCV (%)		7.57	6.90	3.58	34.98	32.76	46.26
GCV (%)		4.45	4.33	2.43	17.85	18.78	21.50
H^2^		0.87	0.88	0.84	0.82	0.82	0.77
GAM		18.31	16.84	10.50	78.67	81.80	99.59

The performance and yield stability of genotypes across environments were assessed using three complementary indices such as Shukla’s stability variance, Wricke’s ecovalence (Wi), and the cultivar superiority index (Pi) (Table 3). A genotype was classified as high-yielding if its mean grain yield exceeded the grand mean (1,394.32 kg ha ⁻ ¹), and as stable if it ranked in the top third (rank ≤ 7 of 21) on at least two of the three parametric stability indices simultaneously. Significant differences were observed among genotypes with respect to both average grain yield and their stability rankings under the three indices. Grain yield means varied widely, with BM22 producing the highest yield (2,086.6 kg ha ⁻ ¹), followed by BP02 (1,771.8 kg ha ⁻ ¹), MM142 (1,761.5 kg ha ⁻ ¹), and BP15 (1,753.3 kg ha ⁻ ¹). According to genotypes BP15, IT99K573-1–1, and MM65 exhibited the lowest variance estimates, indicating superior yield stability across environments. Similarly, the cultivar superiority index identified BP15 (rank 1), BL22 (rank 2), and BP02 (rank 3) as superior performers, combining high yield potential with wide adaptability. Consistent results were obtained using Wricke’s ecovalence, with BP15, IT99K573-1–1, and MM65 again exhibiting the lowest values, confirming their yield stability across environments. For BP15 (ranked 1st on all three stability indices with a mean GY of 1,753.3 kg ha ⁻ ¹), highlighting its practical value as a broadly adapted genotype suitable for recommendation across all six environments tested. For BM22 (highest mean GY at 2,086.6 kg ha ⁻ ¹ but ranked 21st for stability), indicating its suitability for deployment in high-potential, higher-rainfall environments specifically. Other genotypes, including ML04, ML26, MM156, and MM98 displayed both low yields (<1,100 kg ha ⁻ ¹) and poor stability ranks, making them less promising for breeding. In addition to exhibiting stable grain yield across environments, several genotypes showed clear dual-purpose potential by simultaneously producing high and consistent grain and fodder yields (Table 2; S1Table). Among these, BM22, BP02, and BP15 were particularly notable. BM22 combined early flowering (about 30 days) with the highest average grain yield (2,091.9 kg ha ⁻ ¹) and significant fodder production (3,924.7 kg ha ⁻ ¹). Likewise, BP02 expressed early flowering (31.9 days) alongside strong grain (1,775.6 kg ha ⁻ ¹) and fodder yields (3,716.2 kg ha ⁻ ¹). BP15 also performed consistently well, exhibiting high yield stability with a mean grain yield of 1,733.5 kg ha ⁻ ¹, while producing 3,206.9 kg ha ⁻ ¹ of fodder.

**Table 3:** Stability indices computed for grain yield of 21 extra-early cowpea genotypes tested across six environments.

Geno	Mean-GY	Rank	Shukla	Rank	Superiority	Rank	Wricke’s	Rank
65B5080	1485.532	9	87368.67	11	72.42618	10	1257301	11
BL18	1265.046	12	42390.55	7	66.87826	12	646883.7	7
BL22	1593.287	6	25611.34	4	101.4096	2	419165.8	4
BM12	1432.581	11	138400.9	16	58.07132	13	1949881	16
BM13	1516.667	8	43363.68	8	88.97287	6	660090.5	8
BM21	1518.519	7	101403.6	14	80.47349	8	1447775	14
BM22	2086.632	1	335865.3	21	91.37675	5	4629755	21
BM23	1229.225	15	145675	17	54.78446	14	2048601	17
BP02	1771.759	2	128808.8	15	97.94838	3	1819702	15
BP15	1753.328	4	14394.7	1	109.1785	1	266940	1
BP18	1439.236	10	38725.33	5	78.721	9	597141.4	5
IT99K-573_1_1	1075.289	17	15556.09	2	45.06954	16	282701.8	2
ML04	983.5648	21	157603.9	19	36.12553	19	2210494	19
ML23	1054.745	19	61817.99	9	41.4362	17	910541.8	9
ML26	1176.968	16	284986	20	22.77456	21	3939251	20
MM141	1610.301	5	41316.79	6	87.2704	7	632311.2	6
MM142	1761.458	3	98745.9	13	95.57244	4	1411706	13
MM156	1022.454	20	88706.07	12	33.33247	20	1275451	12
MM65	1262.616	13	22287.08	3	69.71978	11	374050.9	3
MM98	1062.674	18	155610	18	39.51981	18	2183433	18
UAM09_1055_6	1256.481	14	75914.07	10	53.90454	15	1101846	10

### Correlations among traits

**Fig 2:**
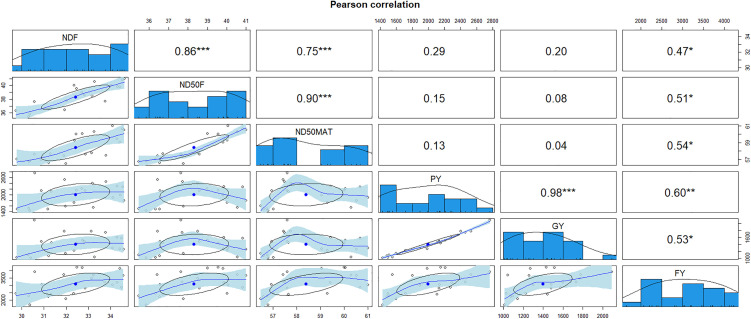
Pearson correlation coefficients among phenological traits pod yield, grain yield, and fodder yield of 21 extra-early cowpea genotypes across six environments in Niger.

Correlation analysis showed significant positive relationships among phenological and yield traits (Fig 2). In particular, days to 50% flowering (ND50F) was strongly correlated with days to first flowering (NDF; r = 0.86) and days to 50% maturity (ND50MAT; r = 0.90), reflecting strong synchrony among earliness traits. A strong correlation was observed between pod yield and grain yield (r = 0.98), indicating that genotypes producing more pods also tend to achieve higher grain output. Although correlations between GY and phenological traits were positive but weak, this implies that earliness does not reduce grain productivity.

Fodder yield showed moderate positive correlations with phenological traits, including NDF (r = 0.47), ND50F (r = 0.51), and ND50MAT (r = 0.54), indicating that genotypes with longer growth duration tend to accumulate greater vegetative biomass. Furthermore, fodder yield was significantly related to both grain yield (r = 0.53) and pod yield (r = 0.60), implying that some genotypes can combine high grain production with substantial fodder biomass. This highlights their dual-purpose suitability, particularly under the resource-limited conditions typical of semi-arid environments.

### Stability of genotypes based on GGE

**Fig 3:**
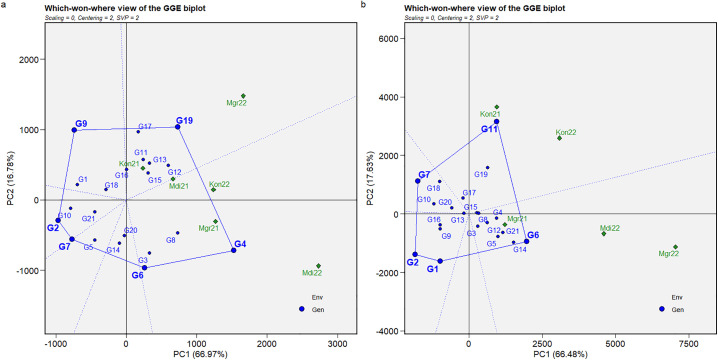
Which-won-where GGE biplot for 21 extra-early cowpea genotypes across six Nigerien environments, showing (a) grain yield and (b) fodder yield. Blue labels indicate genotypes, green labels indicate environments (Kon21/22 = Konni 2021/2022; Mdi21/22 = Maradi 2021/2022; Mag21/22 = Magaria 2021/2022). Polygons highlight the top-performing genotypes within each sector, while environments are organized into distinct mega-environments.

The Which-Won-Where pattern of the GGE biplot was used to examine the specific adaptation of genotypes for (a) grain yield and (b) fodder yield across test environments. For grain yield, the first two principal components accounted for 83.75% of the total genotype × environment interaction (GEI), indicating that the biplot reliably captured the underlying interaction structure. Polygon vertices identified BP02, BM22, BM12, ML26, ML04, and MM98 as the winning genotypes, each representing superior performance within distinct environmental sectors. BP02 and BM22 were closely associated with Mgr22 and Mdi22, respectively, suggesting their suitability for these environments. On the other hand, ML26 and ML04 were positioned as the most responsive genotypes in Kon21, while MM98 showed environment-specific adaptation but comparatively lower mean grain yield than BP02 and BM22. For fodder yield, the first two principal components accounted for 84.41% of the total genotype × environment interaction, supporting the reliability of the biplot-based interpretation. The polygon views revealed BM13, BM12, ML23, ML04, and MM98 as winning genotypes. Genotype BM13 was the top performer in Kon21 and Kon22, while BM12 excelled in Mdi22. These inferences are based on the proximity of each genotype to the respective environments in the biplot (Fig 3).

**Fig 4:**
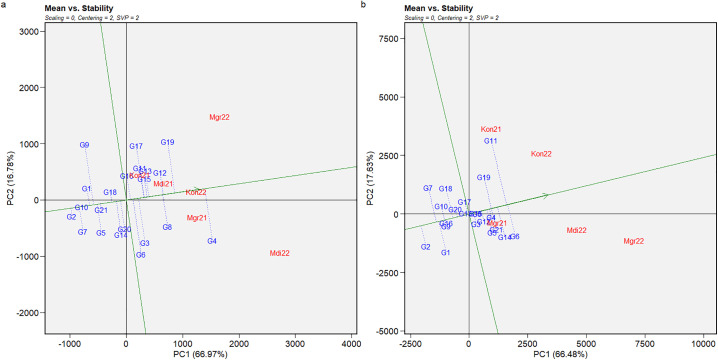
GGE biplot analysis showing mean performance versus stability for (a) grain yield and (b) fodder yield of 21 extra-early cowpea genotypes evaluated across six environments in southeastern Niger. Genotype codes are shown in blue, while environments are labeled in red. The genotypes are: G1: ML23; G2: ML04; G3: 65B5080; G4: BM22; G5: BM23; G6: BM12; G7: MM98; G8: MM142; G9: ML26; G10: MM156; G11: BM13; G12: BP15; G13: BL22; G14: BL18; G15: MM141; G16: BP18; G17: BM21; G18: MM65; G19: BP02; G20: UAM09_1055_6; G21: IT99K573_1_1.

Fig 4 presents the GGE biplot illustrating mean performance in relation to stability. For grain yield (Fig 4a) genotype BM22 showed the highest average performance and exhibited good stability across the six environments, as evidenced by its extended projection along the average environment axis (AEA). Overall, BM22 ranked highest in mean grain yield, followed by BP02, BP15, and MM142, while MM141 was identified as the most stable genotype due to its minimal deviation from the AEA. Conversely, ML04, MM98, and MM156 consistently showed lower grain yields across the evaluated environments. For fodder yield (b), BM12 and BM13 showed the highest mean fodder yield, whereas MM142 and MM141 were the most stable, with the shortest perpendicular mark on the axis. BL18 and BP02 also had high mean yield values but were less stable, as indicated by the longer perpendicular marks on the axis (Fig 4).

### Selection of best-performing genotypes using MGIDI index

**Fig 5:**
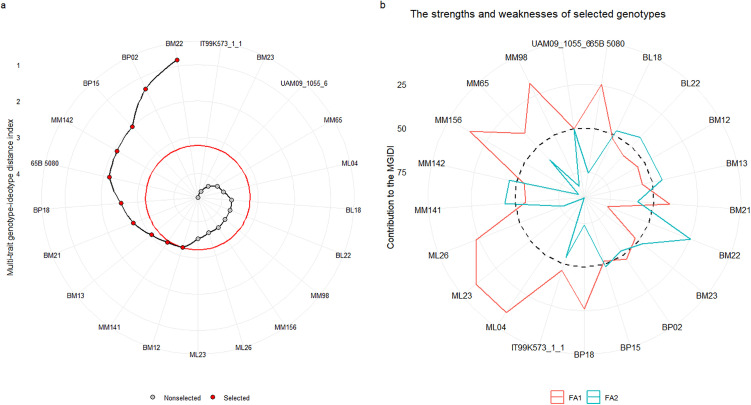
Multi-trait Genotype-Ideotype Distance Index (MGIDI) analysis showing ranking of 21 extra-early cowpea genotypes based on distance from the ideotype (a) and strengths and weaknesses profiles of selected genotypes across multiple traits (FA1 and FA2 represent factor scores from principal component analysis) (b).

The MGIDI (Multi-trait Genotype–Ideotype Distance Index) was applied to identify superior genotypes) by considering all the measured traits. Genotypes were ranked according to their MGIDI values, with those exhibiting lower distances to the ideotype considered more desirable. In the graphical representation (Fig. 5a), genotypes with lower MGIDI values are positioned closer to the center of the plot, whereas those with higher values appear toward the periphery. Genotype selection was based on MGIDI thresholds, indicated by the red dots. Applying this approach, BM22, BP02, BP15, MM142, 65B5080, BP18, BM21, BM13, MM141, and BM12 were identified as the most promising genotypes (Fig. 5a). Notably, none of the check varieties ranked among the selected group, indicating that the tested genotypes surpassed the checks in overall multi-trait performance. These selected lines therefore represent strong candidates for the simultaneous improvement of phenological and productivity traits. Factor analysis was performed to evaluate the strengths and weaknesses of genotypes selected based on the MGIDI index. Two factors, FA1 and FA2, were retained, which together accounted for 88.15% of the total variation. FA1, which accounted for 56.35% of the variation, was mainly associated with phenological traits such as days to first flowering, days to 50% flowering, and days to 50% maturity. FA2 explained 31.80% of the variance and was mainly related to productivity traits, namely pod yield, grain yield, and fodder yield (S2 Table). The MGIDI-based selection achieved a success rate of approximately 44%, corresponding to favorable selection differentials in three out of the six evaluated traits (S3 Table). The contributions of each factor to cultivar performance are shown in Fig. 5b, suggesting the strengths and weaknesses of the genotypes. In this plot, the position of factor contributions—either closer to the center or toward the edge of the circle—reflects their influence on each genotype. Dashed reference lines show the average performance for each factor. Higher factor scores closer to the center indicate relative weaknesses, whereas lower scores located farther from the center denote strengths for a given genotype.

## Discussion

Plant breeding programs increasingly focus on identifying genotypes that simultaneously express multiple favorable traits, rather than excelling in a single trait. To achieve this goal, a wide range of selection indices and multi-trait methods have been developed and applied to efficiently identify superior genotypes [37]. In arid and semi-arid regions, where crop production is often limited by irregular rainfall and poor soil conditions, genetic improvement through selection remains a sustainable and long-term strategy for enhancing yield stability [38]. A core component of this strategy involves the evaluation of diverse germplasm across contrasting environments to identify genotypes with either broad or specific adaptation [39]. Breeding for consistent production requires testing different varieties in a wide range of environments to identify superior genotypes with broad adaptation across environments or specialized adaptation to specific conditions, due to genotype x environment interactions [40]. In this study, extra-early cowpea genotypes exhibited significant genetic variability when evaluated across multiple environments in southeastern Niger. The highly significant effects of genotype, environment, and GEI reflect the complex nature of cowpea adaptation under heterogeneous agro-ecological conditions. These results are consistent with previous studies reporting strong environmental modulation of cowpea performance, especially under rainfed systems where rainfall patterns and soil fertility vary widely between locations and seasons [17,41,42]. The wide range observed for both phenological, and yield-related traits suggests the presence of significant exploitable genetic diversity within the evaluated genotypes. High heritability estimates—particularly for yield traits (H² = 0.82 for PY and GY; 0.77 for FY)—indicate that a large proportion of phenotypic variance is attributable to genetic factors, making selection effective. High GAM values (78.67–99.59% for yield traits) further imply that additive gene action predominates, enabling gains through simple selection strategies. Comparable findings in cowpea and other legumes have associated high heritability and genetic advance with the predominance of additive gene effects, thereby favouring genetic gain through conventional breeding approaches [38,43–49].

The highly significant variation (p < 0.001) observed in the analysis of variance for all six traits indicates substantial genetic diversity among the genotypes evaluated in this study. The plant breeding correlation matrix is an important method to assess the relationship between two or more traits [48,49]. The strong positive association between pod yield and grain yield confirms that pod production is a major determinant of grain productivity, as noted in earlier studies [44,50]. Extra-early maturity provides drought escape by completing grain filling before the short rainy season but theoretically limits vegetative growth duration and hence fodder biomass accumulation. Our correlation analysis, however, showed that within our extra-early set, fodder yield was positively associated with phenological traits (NDF: r = 0.47; ND50MAT: r = 0.54), suggesting that relatively later-maturing lines within the extra-early range accumulated more biomass without forfeiting the extra-early classification [44,51,52]. Although all genotypes evaluated were extra-early, the positive correlation between fodder yield and grain yield suggests that certain lines are capable of combining early maturity with substantial biomass production. Identifying extra-early dual-purpose genotypes is especially important for Niger’s farming systems, where both grain for human consumption and fodder for livestock are critical, particularly under the challenges posed by climate variability. Stability analysis revealed clear variation in the performance of genotypes across different environments. Based on the cultivar superiority index (Pi), genotypes BP15, BL22, and BP02 exhibited superior stability, as evidenced by their low Pi values and minimal deviation from maximum yield across environments. These genotypes also contributed least to GEI, indicating consistent performance. This finding is consistent with earlier reports in cowpea and related legumes demonstrating that genotypes with the smallest Pi values consistently combine high mean yield with superior cross-environment stability [53]. Wricke’s ecovalence (Wi) reflects the degree to which each genotype contributes to the total GEI sum of squares; genotypes with small Wi values produce consistent yields relative to the environmental mean, indicating broad adaptation across contrasting conditions [33]. In our study, the identification of genotypes BP15, IT99K-573-1-1, and MM65 as the top performers with very small mean yield deviations across the six environments using Wricke’s ecovalence stability measure. This agrees with [53,54] who reported that the highest yielding cowpea and common bean genotypes showed relatively low ecovalence values. Genotypes with elevated Wi values tend to show environment-specific responses and are generally better suited to particular high-input production conditions [45,55]. In the present study, BM22, ML26, ML04, MM98, and BM23 displayed the highest ecovalence values, suggesting specialized performance that makes them candidates for targeted recommendation rather than broad deployment. The utility of Wi as a selection tool for stable, productive genotypes has been further corroborated in faba bean by Abou-Khater et al [56], lending additional support to the approach applied here. The contrasting profile of BM22—recording the highest mean grain yield (2,086.6 kg ha ⁻ ¹) yet ranking last (21st) for both Shukla’s variance and Wricke’s ecovalence—reflects specific rather than broad adaptation. BM22 was identified as a winning genotype in the high-rainfall environments of Maradi (2022) and Magaria (2022) in Which-Won-Where biplot, but its performance declined markedly under lower-rainfall or unfertilized condition. This pattern is consistent with a resource-responsive genotype that exploits favorable conditions efficiently but lacks the physiological buffering capacity for stable performance across heterogeneous environments. From a breeding perspective, BM22 may be of value for targeted deployment in high-rainfall zones of southeastern Niger but is not suited for broad varietal recommendation. The significant genotype, environment, and GEI effects observed in this study further emphasize the necessity of multi-environment testing when targeting yield improvement in cowpea. Comparable findings have been widely reported in cowpea and other legumes, highlighting the dominant role of environmental variability and GEI in yield expression [45,46,57–59], and similar patterns have been reported in other major cereal crops; for instance, combined analysis of variance across environments in wheat multi-environment trials revealed highly significant genotype, environment, and genotype × environment interaction effects, underscoring the broad relevance of GEI analysis across cropping systems [60]. The large proportion of total variation attributed to environmental effects underscores the importance of evaluating genotypes across diverse locations to reliably identify broadly adapted and stable lines. In summary, The GGE biplot analysis provided an effective graphical method for visualizing GEI patterns and identifying both superior genotypes and representative testing environments [61,62]. The significant influence of GEI observed across traits suggests the need to incorporate stability analysis into genotype evaluation [54,63,64]. Similar studies have shown that GGE biplot analysis effectively discriminates genotypes and environments for yield and related traits [45,65]. However, selecting genotypes based on multiple traits simultaneously remains challenging when traits are numerous and interrelated. To address this challenge, multivariate methods such as principal component analysis, factor analysis, and cluster analysis are frequently employed [66]. More recently, the MGIDI (multi-trait genotype–ideotype distance index) has emerged as a robust tool for integrating multiple traits into a single selection criterion [20]. In the present study, the MGIDI index successfully identified BM22, BP02, BP15, MM142, 65B5080, BP18, BM21, BM13, MM141, and BM12 as superior genotypes, effectively combining earliness with high grain and fodder yields. Notably, these genotypes flowered and matured earlier than the checks while maintaining superior productivity, highlighting their breeding value. The combination of early maturity and dual-purpose performance is particularly advantageous under Niger’s short and unpredictable rainy seasons. Early harvest reduces exposure to late-season drought and insect pressure, while the availability of fodder shortly after grain harvest supports livestock feeding during critical periods. The successful application of the MGIDI index in this study aligns with its increasing use in other crops such as rice [67], maize [68], yam [69], sweat potato [25], and sorghum [70], underscoring its broad applicability in modern breeding programs. The contrasting fertilization regimes between 2021 (no fertilizer applied) and 2022 (NPK 15:15:15 at 6 g per plant) constituted a deliberate source of environmental variation designed to reflect the range of agronomic management conditions encountered in farmer fields in southern Niger, where fertilizer use is variable and often limited by cost. The significant year effect and GEI observed in this study are therefore partly attributable to differential genotype responses to nitrogen and phosphorus availability, in addition to the large inter-annual rainfall differences (Konni 2021: 323.5 mm vs. Magaria 2022: 741.4 mm). Genotypes that maintained relative stability under both fertilized and unfertilized conditions—notably BP15, MM65, and IT99K573-1–1 (lowest Shukla, Wi, and Pi values)—may possess greater buffering capacity against variable soil nutrient supply, a particularly valuable trait for resource-poor smallholder farming systems in Niger.

These results have direct implications for INRAN’s cowpea breeding pipeline. The ten selected genotypes are candidates for submission to INRAN’s national performance, the formal prerequisite for variety release in Niger. Cross-border multi-environment evaluation in collaboration with IITA-West Africa and national programs in Mali and Burkina Faso—where similar semi-arid production conditions prevail—would further validate their performance across a broader range of agroecological zones. Participatory variety selection (PVS) trials with smallholder farmers in Niger are recommended to validate farmer acceptance of these dual-purpose lines before formal recommendation.

## Conclusion

This study demonstrates that extra-early maturity and dual-purpose productivity are compatible traits in cowpea a finding of direct relevance for climate adaptation in the Sahel, where shortening rainy seasons increasingly favor short-duration crops. The ten genotypes identified through combined stability analysis and the MGIDI index (BM22, BP02, BP15, MM142, 65B5080, BP18, BM21, BM13, MM141, and BM12) are promising candidates for further evaluation. However, given that trials were conducted over two growing seasons across six site-year combinations, multi-season and multi-location validation across additional agroecological zones is required before formal variety release recommendations can be made. Future research should prioritize participatory variety selection with smallholder farmers in the target regions to ensure that performance advantages translate into adoption outcomes, and to validate farmer preferences for grain quality, fodder quantity, and compatibility with local agronomic practices. These efforts, combined with cross-border collaboration, will be essential for maximizing the impact of these genotypes on food and feed security in Niger and the wider Sahel.

## Supporting information

S1 Table Mean performance of the 21 genotypes evaluated across the environment. (XLSX)

S2 Table Factorial loadings, communalities, uniqueness for genotypes based on the multi-trait genotype–ideotype distance index (Bold values are traits with high contribution to each component). (XLSX)

S3 Table Selection gain for mean performance across the environments based on the MGIDI values. (XLSX)
