# Supplementary material for: Stability of grain and fodder yields in extra-early cowpea genotypes in Southeastern Niger
Source: PLoS One. 2026 Jul 28;21(7):e0354617. doi: 10.1371/journal.pone.0354617 (PMC13412050; doi:10.1371/journal.pone.0354617)
Supplement: Attachment — Submitted filename: Response.pdf [file pone.0354617.s004.pdf]

Dear Editors-in-Chief,

I am writing to submit the revised version of the manuscript titled "***Stability of grain and fodder yields in extra-early cowpea genotypes in Southeastern Niger***." following your comments.

We acknowledge and appreciate your comments regarding the text overlap identified between this paper and previous works. We sincerely apologize for this oversight.

In response to your comments, we have carefully and thoroughly revised the manuscript to address all concerns (all overlapping text outside the Methods section) has been fully rephrased. We believe that these revisions have substantially improved the manuscript and now fully comply with the *PLOS ONE* submission guidelines.

Thank you for giving us the opportunity to revise this work.

Thank you for your time and consideration and look forward to your further evaluation.

Sincerely,

Abdoul Moumouni Iro Sodo

abdoulmoumouniirosodo@gmail.com
