# Supplementary material for: Stability of grain and fodder yields in extra-early cowpea genotypes in Southeastern Niger
Source: PLoS One. 2026 Jul 28;21(7):e0354617. doi: 10.1371/journal.pone.0354617 (PMC13412050; doi:10.1371/journal.pone.0354617)
Supplement: Attachment — Submitted filename: Response to Reviewers.pdf [file pone.0354617.s005.pdf]

Dear Editors-in-Chief,

I am writing to submit the revised version of the manuscript titled " ***Stability of grain and fodder yields in extra-early cowpea genotypes in southeastern Niger*** **"** following reviewers' comments and suggestions received from PLOS ONE Journal.

We really appreciate the editor and the reviewers' insightful comments and suggestions. We have carefully considered all suggestions and have revised the manuscript accordingly. Below, we provide a point-by-point response to each comment. All changes made to the manuscript are indicated in the track-changes version submitted alongside this letter.

## I. Journal Requirements:

**PLOS ONE Style Formatting**

*Please ensure that your manuscript meets PLOS ONE's style requirements, including those for file naming.*

**Response:**

The manuscript has been carefully formatted to comply with all *PLOS ONE* style guidelines. All submitted files—including the main manuscript, figures, and supplementary tables—have been renamed following *PLOS ONE* file-naming guide.

**Text Overlap with Popoola et al. (2024)**

*Minor overlapping text detected with: Popoola et al. (2024) 'Assessing the Impact of Genotype-by-Environment Interactions on Agronomic Traits in Elite Cowpea Lines across Agro-Ecologies in Nigeria' (https://doi.org/10.3390/agronomy14020263). Please cite all sources and rephrase any duplicated text outside the Methods section.*

**Response:**

We acknowledge this concern. Upon careful review, the overlapping texts were found in the Introduction and Discussion sections, where similar background statements on cowpea GEI and stability had been utilized. All such passages have been fully rephrased in our own words. Popoola et al. (2024) was already cited in our manuscript (reference [46]) and continues to be cited appropriately where relevant. The overlap has been fully resolved in the revised manuscript.

**Requirement 3: Field Site Permits**

*Please provide additional information regarding the permits obtained for field work and the full name of the authority that approved field site access***.**

**Response:** Field trials were conducted at experimental stations under the direct institutional mandate of the Institut National de la Recherche Agronomique du Niger (INRAN), the national

agricultural research institute operating under the authority of the Ministry of Agriculture of Niger. No additional regulatory permits were required for conducting agronomic field experiments at INRAN-managed research stations, as institutional authorization is embedded within INRAN's research mandate and field access is governed by the institute's annual research planning process.

**Amended Funding Statement**

*Please declare all funding or sources of support received and include: 'There was no additional external funding received for this study.*

**Response:** The Funding Statement has been amended as follows: 'This research was supported by the SAPEP grant. There was no additional external funding received for this study. This amended statement is provided in our cover letter for the editorial office to update the online submission form accordingly.

**Role of Funder Statement**

*Please state what role the funders played in the study*

**Response:** The funders had no role in study design, data collection and analysis, decision to publish, or preparation of the manuscript.

**Figure Quality — Figures 1–5**

*Please upload new copies of Figures 1 to 5, as the detail is not clear*

**Response:** All five figures have been improved and saved in TIFF format.

**<u>II. REVIEWER 1 COMMENTS</u>:**

**Abstract — Quantitative Values for Variation Sources**

*The abstract would benefit from including explicit values for the extent of yield variation or the percentage of total variation explained by genotype, environment, and GEI to give readers a quantitative sense of stability.*

**Response:** The Abstract has been revised to include key quantitative information derived from ANOVA. We now state that environmental effects had the highest mean squares for all traits ($p < 0.001$), followed by genotype and G×E interaction effects. Broad-sense heritability ranged from 0.77 (FY) to 0.88 (ND50F), and the genetic advance as percentage of mean was high for yield traits (GAM = 78.67% for PY, 81.80% for GY, and 99.59% for FY). Mean grain yield across all genotypes and environments was 1,394.32 kg $ha^{-1}$, and the coefficient of variation ranged from 26.59% (GY) to 40.63% (FY) (Lines15-22).

**Introduction — Conciseness and Redundancy**

*Paragraphs can be more concise. Condense duplicated ideas about drought and biotic stress*

**Response:** We have revised the Introduction to consolidate overlapping statements on drought, low soil fertility, Striga infestation, and insect pests, which were discussed redundantly across multiple paragraphs (lines42-47).

**Study Hypothesis**

*The objective is clearly stated, but it would help to specify the hypothesis — i.e., whether dual-purpose performance can be achieved without penalizing early maturity.*

**Response:** An explicit hypothesis statement has been added at the end of the Introduction: among the extra-early cowpea genotypes developed at INRAN, it is possible to identify lines that combine high grain and fodder yield with stability across contrasting environments without compromising the phenological advantage of extra-early maturity—a key adaptive trait in Niger's short and erratic rainy seasons (lines75-79).

**Criteria for Environment Selection**

*Include the criteria for selecting the six environments (e.g., agroecological differences, rainfall pattern, soil type).*

**Response:** The three sites were selected to represent the main agro-climatic zones of southern Niger where cowpea is commercially produced. The soils across the experimental sites were predominantly sandy, although some variation in texture was observed. Maradi soil consisted of 72% sand, 11% silt, and 17% clay, while Magaria soil was more sandy, with 88% sand, 4% silt, and 8% clay. whereas Konni soils were characterized by 70% sand, 10.2-30% silt, and 15-20% clay. Soil organic carbon content was generally low across all sites, indicating poor soil fertility. Values were 0.30% at Maradi, 0.27% at Magaria, and 0.15% at Konni. Soil pH ranged from slightly acidic to near neutral, with values of 5.4 at Maradi, 5.8 at Magaria, 5.5 at Konni (lines93-103).

**Balanced Data and Missing Values**

*Explicitly state whether data were balanced across environments, or how missing data (if any) were handled.*

**Response:** The dataset was fully balanced across all six environments, except in Maradi (2021) no data on fodder yield was recorded.

**MGIDI — Trait Weighting and Rescaling Direction**

*The MGIDI methodology is presented mathematically but not operationally described. Please include more detail on trait weighting and rescaling direction (which traits were desired higher or desired lower).*

**Response:** We have expanded the MGIDI subsection in the Methods to provide full operational clarity. The ideotype was defined as a hypothetical genotype combining: maximum grain yield (GY), pod yield (PY), and fodder yield (FY)—traits for which higher values are desired ($\eta nj$ = 100, $\phi nj$ = 0 in the rescaling formula); and minimum days to first flowering (NDF), days to 50% flowering (ND50F), and days to 50% maturity (ND50MAT)—traits for which lower values are desired ($\eta nj$ = 0, $\phi nj$ = 100), consistent with the extra-early maturity breeding objective (lines184-189).

**Rationale for Using Multiple Stability Indices**

*A sentence explaining why these specific stability indices were chosen (e.g., complementary strengths) would improve methodological transparency.*

**Response:** We have added the following rationale to the Statistical Analysis subsection: These stability statistics were selected for their complementary strengths: Shukla's stability variance and Wricke's ecovalence quantify static (biological) stability by measuring variance in genotype performance across environments; the cultivar superiority index (Pi) simultaneously integrates yield level and stability by penalizing deviation from the highest-yielding genotype per environment; and the GGE biplot provides visual diagnostic capability for identifying mega-environments and comparing genotypes graphically (lines143-149).

**Tables 2–3 — Biological Interpretation**

*Consider summarizing Tables 2–3 more narratively — focus on biological rather than statistical interpretation.*

**Response:** The Results section accompanying Tables 2 and 3 has been revised to emphasize agronomic rather than statistical significance. For BP15 (ranked 1st on all three stability indices with a mean GY of 1,753.3 kg $ha^{-1}$), we now highlight its practical value as a broadly adapted genotype suitable for recommendation across all six environments tested. For BM22 (highest mean GY at 2,086.6 kg $ha^{-1}$ but ranked 21st for stability), indicating its suitability for deployment in high-potential, higher-rainfall environments specifically, rather than merely reporting the statistical rank contrast. Similarly, for genotypes with both low yield and poor stability (ML04, ML26, MM156, MM98), we now briefly discuss their limited breeding value and reduced priority for advancement (lines290-294).

**Figure Resolution**

*Figures (especially GGE biplots and violin plots) need higher resolution.*

**Response:** All figures have been regenerated at high resolution — see Response to Journal Requirement.

**BM22 — High Yield but Poor Stability**

*Discuss briefly the discrepancy between high grain yield and poor stability observed in BM22 — what environmental or genetic factors may underlie such sensitivity?*

**Response:** We have added a focused paragraph in the Discussion: The contrasting profile of BM22—recording the highest mean grain yield (2,086.6 kg $ha^{-1}$) yet ranking last (21st) for both Shukla's variance and Wricke's ecovalence—reflects specific rather than broad adaptation. BM22 was identified as a winning genotype in the high-rainfall environments of Maradi (2022) and Magaria (2022) in the Which-Won-Where biplot , but its performance declined markedly under lower-rainfall or unfertilized conditions. This pattern is consistent with a resource-responsive genotype that exploits favorable conditions efficiently but lacks the physiological buffering capacity for stable performance across heterogeneous environments. From a breeding perspective, BM22 may be of value for targeted deployment in high-rainfall zones of southeastern Niger but is not suited for broad varietal recommendation (lines449-457).

**Discussion — Deeper Cause–Effect Reasoning**

*The discussion is sometimes descriptive; aim for deeper cause–effect reasoning (e.g., link genotype performance to rainfall variation or soil fertility where possible).*

**Response:** The Discussion has been substantially revised to emphasize mechanistic interpretation. The contrasting fertilization regimes between 2021 (no fertilizer applied) and 2022 (NPK 15:15:15 at 6 g per plant) constituted a deliberate source of environmental variation designed to reflect the range of agronomic management conditions encountered in farmer fields in southern Niger, where fertilizer use is variable and often limited by cost. The significant year effect and GEI observed in this study are therefore partly attributable to differential genotype responses to nitrogen and phosphorus availability, in addition to the large inter-annual rainfall differences (Konni 2021: 323.5 mm vs. Magaria 2022: 741.4 mm). Genotypes that maintained relative stability under both fertilized and unfertilized conditions—notably BP15, MM65, and IT99K573-1-1 (lowest Shukla, Wi, and Pi values)—may possess greater buffering capacity against variable soil nutrient supply, a particularly valuable trait for resource-poor smallholder farming systems in Niger (lines483-493).

**Remove Redundant Citations and Overlapping Explanations**

*Remove redundant citations or overlapping explanations of the same concepts (e.g., stability interpretation repeated from Results).*

**Response:** We have reviewed the entire manuscript and removed passages in the Discussion that unnecessarily restated stability definitions or interpretations already provided in the Methods or Results.

**Practical Breeding Implications**

*Include more practical breeding implications: how these genotypes might integrate into current INRAN or regional breeding pipelines*

**Response:** We have added a dedicated paragraph in the Discussion on breeding pipeline integration: These results have direct implications for INRAN's cowpea breeding pipeline. The ten selected genotypes are candidates for submission to INRAN's national performance, the formal prerequisite for variety release in Niger. Cross-border multi-environment evaluation in collaboration with IITA-West Africa and national programs in Mali and Burkina Faso—where similar semi-arid production conditions prevail—would further validate their performance across a broader range of agroecological zones. Participatory variety selection (PVS) trials with smallholder farmers in Niger are recommended to validate farmer acceptance of these dual-purpose lines before formal recommendation (lines494-501).

**Conclusion — Implications Over Results**

*Avoid restating results verbatim in the Conclusion. Focus instead on implications (climate resilience, early adoption, need for participatory trials).*

**Response:** The Conclusion section has been rewritten: this study demonstrates that extra-early maturity and dual-purpose productivity are compatible traits in cowpea a finding of direct relevance for climate adaptation in the Sahel, where shortening rainy seasons increasingly favor short-duration crops. The ten genotypes identified through combined stability analysis and the MGIDI index (BM22, BP02, BP15, MM142, 65B5080, BP18, BM21, BM13, MM141, and BM12) are promising candidates for further evaluation. However, given that trials were conducted over two growing seasons across six site-year combinations, multi-season and multi-location validation across additional agroecological zones is required before formal variety release recommendations can be made. Future research should prioritize participatory variety selection with smallholder farmers in the target regions to ensure that performance advantages translate into adoption outcomes, and to validate farmer preferences for grain quality, fodder quantity, and compatibility with local agronomic practices. These efforts, combined with cross-border collaboration, will be essential for maximizing the impact of these genotypes on food and feed security in Niger and the wider Sahel (lines503-515).

## III. REVIEWER #2 COMMENT

**Criteria for Classifying Genotypes as High-Yielding and Stable**

*The criteria used to classify genotypes as both high yielding and stable should be explained more clearly. A summary table integrating yield performance and all stability metrics would improve interpretation.*

**Response:** A paragraph has been added to the Results: a genotype was classified as high-yielding if its mean grain yield exceeded the grand mean (1,394.32 kg $ha^{-1}$), and as stable if it ranked in the top third (≤ 7 out of 21) on at least two of the three stability indices simultaneously (lines279-281). The existing Table 2 has been already to present mean GY alongside all three stability ranks in a single integrated table.

**Environmental Characterization**

*The environmental characteristics of the six testing sites are not sufficiently described. Additional information on rainfall, soil properties, and climatic conditions would help readers understand sources of GEI.*

**Response:** We have added a dedicated environmental characterization: The three sites were selected to represent the main agro-climatic zones of southern Niger where cowpea is commercially produced. The soils across the experimental sites were predominantly sandy, although some variation in texture was observed. Maradi soil consisted of 72% sand, 11% silt, and 17% clay, while Magaria soil was more sandy, with 88% sand, 4% silt, and 8% clay. whereas Konni soils were characterized by 70% sand, 10.2-30% silt, and 15-20% clay. Soil organic carbon content was generally low across all sites, indicating poor soil fertility. Values were 0.30% at Maradi, 0.27% at Magaria, and 0.15% at Konni. Soil pH ranged from slightly acidic to near neutral, with values of 5.4 at Maradi, 5.8 at Magaria, 5.5 at Konni (lines93-103).

**Limited Scope — Two Years and Six Environments**

*The study was conducted across only two years and six environments. Broader cultivar release recommendations should be made cautiously until lines are validated across additional agroecological zones and seasons.*

**Response:** We fully agree. This limitation is now explicitly acknowledged in both the Discussion and Conclusion: that trials were conducted over two growing seasons across six site-year combinations, multi-season and multi-location validation across additional agroecological zones is required before formal variety release recommendations can be made (lines531-543).

**Grain–Fodder Yield Relationships and Trade-offs**

*The discussion of grain and fodder yield relationships could be strengthened. Potential trade-offs between early maturity, biomass accumulation, and drought escape deserve deeper consideration.*

**Response:** We have added a paragraph in the Discussion specifically addressing these physiological trade-offs. Extra-early maturity provides drought escape by completing grain filling before the end of the short rainy season but theoretically limits vegetative growth duration and hence fodder biomass accumulation. Our correlation analysis, however, showed that within our extra-early set, fodder yield was positively associated with phenological traits (NDF: r = 0.47; ND50MAT: r = 0.54), suggesting that relatively later-maturing lines within the extra-early range accumulated more biomass without forfeiting the extra-early classification (lines419-425).

**Rationale for Multiple Stability Statistics**

*The rationale for using multiple stability approaches simultaneously should be explained more clearly, including a brief comparison of strengths and limitations.*

**Response:** Please see also response to reviewer #1. The revised Methods now include: These stability statistics were selected for their complementary strengths: Shukla's stability variance and Wricke's ecovalence quantify static (biological) stability by measuring variance in genotype performance across environments; the cultivar superiority index (Pi) simultaneously integrates yield level and stability by penalizing deviation from the highest-yielding genotype per environment; and the GGE biplot provides visual diagnostic capability for identifying mega-environments and comparing genotypes graphically( lines143-149).

**Fertilizer Management Difference Between Years as Source of GEI**

*Fertilizer management differed between years (no fertilizer in 2021; NPK applied in 2022). This management difference may have contributed to GEI and should be discussed.*

**Response:** We thank the reviewer for raising this important point: The contrasting fertilization regimes between 2021 (no fertilizer applied) and 2022 (NPK 15:15:15 at 6 g per plant) constituted a deliberate source of environmental variation designed to reflect the range of agronomic management conditions encountered in farmer fields in southern Niger, where fertilizer use is variable and often limited by cost. The significant year effect and GEI observed

in this study are therefore partly attributable to differential genotype responses to nitrogen and phosphorus availability, in addition to the large inter-annual rainfall differences (Konni 2021: 323.5 mm vs. Magaria 2022: 741.4 mm). Genotypes that maintained relative stability under both fertilized and unfertilized conditions—notably BP15, MM65, and IT99K573-1-1 (lowest Shukla, Wi, and Pi values)—may possess greater buffering capacity against variable soil nutrient supply, a particularly valuable trait for resource-poor smallholder farming systems in Niger (lines483-493).

**MGIDI — Ideotype Definition and Selection Intensity**

*The choice of traits included in the ideotype and the justification for the 50% selection intensity should be explained in greater detail to enhance reproducibility.*

**Response:** The MGIDI subsection has been expanded: The ideotype was defined as a hypothetical genotype combining: maximum grain yield (GY), pod yield (PY), and fodder yield (FY)—traits for which higher values are desired ($\eta nj$ = 100, $\phi nj$ = 0 in the rescaling formula); and minimum days to first flowering (NDF), days to 50% flowering (ND50F), and days to 50% maturity (ND50MAT)—traits for which lower values are desired ($\eta nj$ = 0, $\phi nj$ = 100), consistent with the extra-early maturity breeding objective (lines184-189).

**Language Editing**

*Minor language editing is recommended. The Abstract statement regarding 'users' preferences, future deployment as new varieties or in developing improved varieties' should be revised for clarity.*

**Response:** The manuscript has been carefully proofread and corrected throughout. The specific Abstract sentence has been revised to: These lines should be further evaluated across diverse agroecological zones to confirm their adaptability and alignment with farmer preferences, prior to deployment as released varieties or as parents in cowpea improvement programs targeting climate-resilient, dual-purpose productivity in the Sahel (lines24-27).

**Practical Breeding Implications**

*The discussion focuses primarily on statistical stability. Elaborate on how the selected genotypes could be incorporated into breeding programs or variety release pipelines in Niger and neighboring countries.*

**Response:** Please see response to Reviewer #1: These results have direct implications for INRAN's cowpea breeding pipeline. The ten selected genotypes are candidates for submission to INRAN's national performance, the formal prerequisite for variety release in Niger. Cross-border multi-environment evaluation in collaboration with IITA-West Africa and national programs in Mali and Burkina Faso—where similar semi-arid production conditions prevail—would further validate their performance across a broader range of agroecological zones. Participatory variety selection (PVS) trials with smallholder farmers in Niger are recommended to validate farmer acceptance of these dual-purpose lines before formal recommendation (lines494-501).

We believe the revised manuscript is now substantially improved in scientific depth, methodological transparency, and practical relevance, and we look forward to the Editor's further consideration.

We look forward to hearing from you soon.

Sincerely,

Abdoul Moumouni Iro Sodo
abdoulmoumouniirosodo@gmail.com
