# Supplementary material for: Stability of grain and fodder yields in extra-early cowpea genotypes in Southeastern Niger
Source: PLoS One. 2026 Jul 28;21(7):e0354617. doi: 10.1371/journal.pone.0354617 (PMC13412050; doi:10.1371/journal.pone.0354617)
Supplement: Attachment — Submitted filename: Response_to_Reviewers_auresp_2.pdf [file pone.0354617.s006.pdf]

Dear Editors-in-Chief,

I am writing to submit the revised version of the manuscript titled " ***Stability of grain and fodder yields in extra-early cowpea genotypes in southeastern Niger*** **"** following Editor's comments and suggestions.

We really appreciate the editor for the opportunity to further revise our manuscript. We have carefully addressed the additional comment raised. The changes have been incorporated into the revised manuscript and are highlighted in the tracked-changes version.

Journal Requirements

Please review your reference list to ensure that it is complete and correct

Response:

We have reviewed the full reference list and confirm that it is complete and correct. None of the cited references have been retracted. One new reference (Munir et al., 2024, entry #60) has been added in response to the Academic Editor's comment; no other changes were made to the reference list.

Academic Editor Comment

Although the authors have significantly improved the manuscript, the relevant and recent citations are still missing, I would suggest to cite the following relevant citations.

Munir, R., M. Kashif, M.Z. Iqbal and G. Farid. 2024. Stability Analysis of Genotype × Environment Interaction for Wheat Genotypes Through Bayesian and Frequentist Approaches. Pak. J. Agri. Sci., Vol. 61(4), 1013-1021.

Response: We thank the Academic Editor for this suggestion. We have reviewed Munir et al. (2024) and agree that it is relevant, as it also reports highly significant genotype, environment, and genotype × environment interaction effects from analysis of variance in multi-environment trials and compares Bayesian and frequentist approaches for stability analysis. We have cited this reference in the Discussion section, where we compare our GEI findings with reports from other cropping systems, to reinforce that significant G×E effects are a broadly observed across crops and not specific to cowpea (lines462-465).

The full reference has been added to the reference list as entry #60: Munir R, Kashif M, Iqbal MZ, Farid G. 2024. Stability Analysis of Genotype × Environment Interaction for Wheat Genotypes Through Bayesian and Frequentist Approaches. Pak J Agri Sci. 61:1013–1021. https://doi.org/10.21162/PAKJAS/24.276

We believe the revised manuscript is now substantially improved and we look forward to the Editor's further consideration.

Sincerely,

Abdoul Moumouni Iro Sodo
abdoulmoumouniirosodo@gmail.com
