# Supplementary material for: Stability of grain and fodder yields in extra-early cowpea genotypes in Southeastern Niger
Source: PLoS One. 2026 Jul 28;21(7):e0354617. doi: 10.1371/journal.pone.0354617 (PMC13412050; doi:10.1371/journal.pone.0354617)
Supplement: Peer review history

# Author comment: Author response to Decision Letter 0

**DOI:** 10.1371/journal.pone.0354617.r001

13 Jan 2026

Attachment Submitted filename: Response.pdf

# Peer review: Decision Letter 0

**DOI:** 10.1371/journal.pone.0354617.r002

**Authors:** Muhammad Amjad Ali

17 Jun 2026

PONE-D-25-68979

Stability of grain and fodder yields in extra-early cowpea genotypes in Southeastern Niger

PLOS One

Dear Dr. Moumouni,

Thank you for submitting your manuscript to PLOS ONE. After careful consideration, we feel that it has merit but does not fully meet PLOS ONE’s publication criteria as it currently stands. Therefore, we invite you to submit a revised version of the manuscript that addresses the points raised during the review process.

Please submit your revised manuscript by Aug 01 2026 11:59PM. If you will need more time than this to complete your revisions, please reply to this message or contact the journal office at plosone@plos.org. When you're ready to submit your revision, log on to https://www.editorialmanager.com/pone/ and select the 'Submissions Needing Revision' folder to locate your manuscript file.

Please include the following items when submitting your revised manuscript:

- A letter that responds to each point raised by the academic editor and reviewer(s). You should upload this letter as a separate file labeled 'Response to Reviewers'.
- A marked-up copy of your manuscript that highlights changes made to the original version. You should upload this as a separate file labeled 'Revised Manuscript with Track Changes'.
- An unmarked version of your revised paper without tracked changes. You should upload this as a separate file labeled 'Manuscript'.

If you would like to make changes to your financial disclosure, please include your updated statement in your cover letter. Guidelines for resubmitting your figure files are available below the reviewer comments at the end of this letter.

If applicable, we recommend that you deposit your laboratory protocols in protocols.io to enhance the reproducibility of your results. Protocols.io assigns your protocol its own identifier (DOI) so that it can be cited independently in the future. For instructions see: https://journals.plos.org/plosone/s/submission-guidelines#loc-laboratory-protocols. Additionally, PLOS ONE offers an option for publishing peer-reviewed Lab Protocol articles, which describe protocols hosted on protocols.io. Read more information on sharing protocols at https://plos.org/protocols?utm_medium=editorial-email&utm_source=authorletters&utm_campaign=protocols.

As the corresponding author, your ORCID iD is verified in the submission system and will appear in the published article. PLOS supports the use of ORCID, and we encourage all coauthors to register for an ORCID iD and use it as well. Please encourage your coauthors to verify their ORCID iD within the submission system before final acceptance, as unverified ORCID iDs will not appear in the published article. *Only*  the individual author can complete the verification step; PLOS staff *cannot*  verify ORCID iDs on behalf of authors.

We look forward to receiving your revised manuscript.

Kind regards,

Muhammad Amjad Ali, PhD

Academic Editor

PLOS One

**Journal Requirements:**

1. When submitting your revision, we need you to address these additional requirements.

Please ensure that your manuscript meets PLOS ONE's style requirements, including those for file naming. The PLOS ONE style templates can be found at

https://journals.plos.org/plosone/s/file?id=wjVg/PLOSOne_formatting_sample_main_body.pdf and

https://journals.plos.org/plosone/s/file?id=ba62/PLOSOne_formatting_sample_title_authors_affiliations.pdf

2. We noticed you have some minor occurrence of overlapping text with the following previous publication(s), which needs to be addressed:

Assessing the Impact of Genotype-by-Environment Interactions on Agronomic Traits in Elite Cowpea Lines across Agro-Ecologies in Nigeria -  https://doi.org/10.3390/agronomy14020263

In your revision ensure you cite all your sources (including your own works), and quote or rephrase any duplicated text outside the methods section. Further consideration is dependent on these concerns being addressed.

3. In your Methods section, please provide additional information regarding the permits you obtained for the work. Please ensure you have included the full name of the authority that approved the field site access and, if no permits were required, a brief statement explaining why.

4. Please note that PLOS One has specific guidelines on code sharing for submissions in which author-generated code underpins the findings in the manuscript. In these cases, we expect all author-generated code to be made available without restrictions upon publication of the work. Please review our guidelines at https://journals.plos.org/plosone/s/materials-and-software-sharing#loc-sharing-code and ensure that your code is shared in a way that follows best practice and facilitates reproducibility and reuse.

5. Thank you for stating in your Funding Statement:

This research was supported by the SAPEP project as part of grant provided to conduct this study.

Please provide an amended statement that declares *all* the funding or sources of support (whether external or internal to your organization) received during this study, as detailed online in our guide for authors at http://journals.plos.org/plosone/s/submit-now.  Please also include the statement “There was no additional external funding received for this study.” in your updated Funding Statement.

Please include your amended Funding Statement within your cover letter. We will change the online submission form on your behalf.

6. Thank you for stating the following financial disclosure:

This research was supported by the SAPEP project as part of grant provided to conduct this study.

Please state what role the funders took in the study.  If the funders had no role, please state: "The funders had no role in study design, data collection and analysis, decision to publish, or preparation of the manuscript."

If this statement is not correct you must amend it as needed.

Please include this amended Role of Funder statement in your cover letter; we will change the online submission form on your behalf.

7. Please upload a new copy of Figures 1 to 5, as the detail is not clear. Please follow the link for more information:  https://journals.plos.org/plosone/s/figures

8. If the reviewer comments include a recommendation to cite specific previously published works, please review and evaluate these publications to determine whether they are relevant and should be cited. There is no requirement to cite these works unless the editor has indicated otherwise.

[Note: HTML markup is below. Please do not edit.]

Reviewers' comments:

Reviewer's Responses to Questions

**Comments to the Author**

1. Is the manuscript technically sound, and do the data support the conclusions?

The manuscript must describe a technically sound piece of scientific research with data that supports the conclusions. Experiments must have been conducted rigorously, with appropriate controls, replication, and sample sizes. The conclusions must be drawn appropriately based on the data presented.

Reviewer #1: Partly

Reviewer #2: Yes

**********

2. Has the statistical analysis been performed appropriately and rigorously?

Reviewer #1: Yes

Reviewer #2: Yes

**********

3. Have the authors made all data underlying the findings in their manuscript fully available?

The PLOS Data policy requires authors to make all data underlying the findings described in their manuscript fully available without restriction, with rare exception (please refer to the Data Availability Statement in the manuscript PDF file). The data should be provided as part of the manuscript or its supporting information, or deposited to a public repository. For example, in addition to summary statistics, the data points behind means, medians and variance measures should be available. If there are restrictions on publicly sharing data—e.g. participant privacy or use of data from a third party—those must be specified.

Reviewer #1: Yes

Reviewer #2: Yes

**********

4. Is the manuscript presented in an intelligible fashion and written in standard English?

PLOS ONE does not copyedit accepted manuscripts, so the language in submitted articles must be clear, correct, and unambiguous. Any typographical or grammatical errors should be corrected at revision, so please note any specific errors here.

Reviewer #1: Yes

Reviewer #2: Yes

**********

5. Review Comments to the Author

Please use the space provided to explain your answers to the questions above. You may also include additional comments for the author, including concerns about dual publication, research ethics, or publication ethics. (Please upload your review as an attachment if it exceeds 20,000 characters)

**Reviewer #1:**  The study “Stability of grain and fodder yields in extra-early cowpea genotypes in Southeastern Niger” is relevant and original in scope. Evaluating extra-early cowpea genotypes with dual-purpose (grain and fodder) potential is highly pertinent to Niger’s semi-arid regions. The use of multiple complementary stability statistics and particularly the MGIDI index enhances the robustness of genotype selection.

That said, revisions are necessary to improve clarity, interpretation depth, and reproducibility. My comments are following,

•The abstract sufficiently summarizes the key findings. However, it would benefit from including explicit values for the extent of yield variation or the percentage of total variation explained by genotype, environment, and GEI to give readers a quantitative sense of stability.

•The background is comprehensive, but paragraphs can be more concise. Condense duplicated ideas about drought and biotic stress.

•The literature review would benefit from integrating more regionally contextualized studies from Sahelian West Africa (e.g., Niger, Mali, Chad) to strengthen regional relevance.

•The objective is clearly stated, but it would help to specify the hypothesis — i.e., whether dual-purpose performance can be achieved without penalizing early maturity.

•Include the criteria for selecting the six environments (e.g., agroecological differences, rainfall pattern, soil type).

•Explicitly state whether data were balanced across environments, or how missing data (if any) were handled.

•The MGIDI methodology is presented mathematically, but not operationally described. Please include more detail on trait weighting and rescaling direction (for example: which traits were "desired higher" or "desired lower").

•While the use of multiple indices enriches rigor, a sentence explaining why these specific indices were chosen (e.g., complementary strengths) would improve methodological transparency.

•Consider summarizing Tables 2–3 more narratively—focus on biological rather than statistical interpretation.

•Figures (especially GGE biplots and violin plots) need higher resolution.

•Discuss briefly the discrepancy between high grain yield and poor stability observed in BM22 – what environmental or genetic factors may underlie such sensitivity?

•The discussion is sometimes descriptive; aim for deeper cause–effect reasoning (e.g., link genotype performance to rainfall variation or soil fertility where possible).

•Remove redundant citations or overlapping explanations of the same concepts (e.g., stability interpretation repeated from Results).

•Include more practical breeding implications: how might these genotypes integrate into current INRAN or regional breeding pipelines?

•Concise and relevant but avoid restating results verbatim. Focus instead on implications (climate resilience, early adoption, need for participatory trials).

**Reviewer #2:** The manuscript titled “Stability of Grain and Fodder Yields in Extra-Early Cowpea Genotypes in Southeastern Niger” addresses an important breeding objective for West African agriculture by identifying extra-early cowpea genotypes that combine grain yield, fodder yield, and yield stability across multiple environments. The evaluation of 21 genotypes across six environments, together with the use of several stability statistics (Shukla’s variance, Wricke’s ecovalence, cultivar superiority index, GGE biplot, and MGIDI), represents a strong aspect of the study. The identification of promising dual-purpose cowpea lines is particularly relevant for farming systems in Niger where both grain and fodder are critical components of food and livestock security. However, the manuscript would benefit from revision before it can be considered for publication. The following comments are provided to strengthen the scientific quality and presentation of the work.

1.The manuscript identifies ten promising genotypes; however, the criteria used to classify these lines as both “high yielding” and “stable” should be explained more clearly. A summary table integrating yield performance and all stability metrics would improve interpretation.

2.Environmental effects were reported to account for a substantial proportion of the observed variation, yet the environmental characteristics of the six testing sites are not sufficiently described.

3.Additional information on rainfall, soil properties, and climatic conditions would help readers understand the sources of genotype × environment interaction.

4.The study was conducted across only two years and six environments. While the results are encouraging, broader recommendations for cultivar release should be made cautiously until the identified lines are validated across additional agroecological zones and seasons.

5.The discussion of grain and fodder yield relationships could be strengthened. Although positive associations were reported, the potential trade-offs between early maturity, biomass accumulation, and drought escape mechanisms deserve deeper consideration.

6.Several stability statistics were employed, but the rationale for using multiple approaches simultaneously should be explained more clearly. A brief comparison of the strengths and limitations of each stability method would improve the methodological section.

7.The fertilizer management differed between years, with no fertilizer applied in 2021 and NPK fertilizer applied in 2022. This management difference may have contributed to environmental variation and should be discussed as a potential source of genotype × environment interaction.

8.The MGIDI approach is a valuable addition to the study; however, the choice of traits included in the ideotype and the justification for the 50% selection intensity should be explained in greater detail to enhance reproducibility.

9.Minor language editing is recommended throughout the manuscript. Several grammatical inconsistencies, typographical issues, and sentence structure problems were observed. For example, the statement in the Abstract regarding “users’ preferences, future deployment as new varieties or in developing improved varieties” should be revised for clarity and readability.

10.The discussion focuses primarily on statistical stability, whereas practical breeding implications are less developed. It would be useful to elaborate on how the selected genotypes could be incorporated into breeding programs or variety release pipelines in Niger and neighboring countries.

11.Similar research has been recently carried out on the stability of fodder sorghum https://doi.org/10.55627/pbiotech.004.01.1839 that must be cited in this paper in discussion.

**********

6. PLOS authors have the option to publish the peer review history of their article (what does this mean?). If published, this will include your full peer review and any attached files.

If you choose “no”, your identity will remain anonymous but your review may still be made public.

**Do you want your identity to be public for this peer review?**  For information about this choice, including consent withdrawal, please see our Privacy Policy.

Reviewer #1: No

Reviewer #2: **Yes:** Muhammad Amjad Ali

**********

[NOTE: If reviewer comments were submitted as an attachment file, they will be attached to this email and accessible via the submission site. Please log into your account, locate the manuscript record, and check for the action link "View Attachments". If this link does not appear, there are no attachment files.]

To ensure your figures meet our technical requirements, please review our figure guidelines: https://journals.plos.org/plosone/s/figures

You may also use PLOS’s free figure tool, NAAS, to help you prepare publication quality figures: https://journals.plos.org/plosone/s/figures#loc-tools-for-figure-preparation.

NAAS will assess whether your figures meet our technical requirements by comparing each figure against our figure specifications.

# Author comment: Author response to Decision Letter 1

**DOI:** 10.1371/journal.pone.0354617.r003

22 Jun 2026

Dear Editors-in-Chief,

I am writing to submit the revised version of the manuscript titled " Stability of grain and fodder yields in extra-early cowpea genotypes in southeastern Niger " following reviewers’ comments and suggestions received from PLOS ONE Journal.

We really appreciate the editor and the reviewers’ insightful comments and suggestions. We have carefully considered all suggestions and have revised the manuscript accordingly. Below, we provide a point-by-point response to each comment. All changes made to the manuscript are indicated in the track-changes version submitted alongside this letter.

I. Journal Requirements:

PLOS ONE Style Formatting

Please ensure that your manuscript meets PLOS ONE's style requirements, including those for file naming.

Response:

The manuscript has been carefully formatted to comply with all PLOS ONE style guidelines. All submitted files—including the main manuscript, figures, and supplementary tables—have been renamed following PLOS ONE file-naming guide.

Text Overlap with Popoola et al. (2024)

Minor overlapping text detected with: Popoola et al. (2024) 'Assessing the Impact of Genotype-by-Environment Interactions on Agronomic Traits in Elite Cowpea Lines across Agro-Ecologies in Nigeria' (https://doi.org/10.3390/agronomy14020263). Please cite all sources and rephrase any duplicated text outside the Methods section.

Response:

We acknowledge this concern. Upon careful review, the overlapping texts were found in the Introduction and Discussion sections, where similar background statements on cowpea GEI and stability had been utilized. All such passages have been fully rephrased in our own words. Popoola et al. (2024) was already cited in our manuscript (reference [46]) and continues to be cited appropriately where relevant. The overlap has been fully resolved in the revised manuscript.

Requirement 3: Field Site Permits

Please provide additional information regarding the permits obtained for field work and the full name of the authority that approved field site access.

Response: Field trials were conducted at experimental stations under the direct institutional mandate of the Institut National de la Recherche Agronomique du Niger (INRAN), the national agricultural research institute operating under the authority of the Ministry of Agriculture of Niger. No additional regulatory permits were required for conducting agronomic field experiments at INRAN-managed research stations, as institutional authorization is embedded within INRAN's research mandate and field access is governed by the institute's annual research planning process.

Amended Funding Statement

Please declare all funding or sources of support received and include: 'There was no additional external funding received for this study.

Response: The Funding Statement has been amended as follows: This research was supported by the SAPEP grant. There was no additional external funding received for this study. This amended statement is provided in our cover letter for the editorial office to update the online submission form accordingly.

Role of Funder Statement

Please state what role the funders played in the study

Response: The funders had no role in study design, data collection and analysis, decision to publish, or preparation of the manuscript.

Figure Quality — Figures 1–5

Please upload new copies of Figures 1 to 5, as the detail is not clear

Response: All five figures have been improved and saved in TIFF format.

II. REVIEWER 1 COMMENTS:

Abstract — Quantitative Values for Variation Sources

The abstract would benefit from including explicit values for the extent of yield variation or the percentage of total variation explained by genotype, environment, and GEI to give readers a quantitative sense of stability.

Response: The Abstract has been revised to include key quantitative information derived from ANOVA. We now state that environmental effects had the highest mean squares for all traits (p < 0.001), followed by genotype and G×E interaction effects. Broad-sense heritability ranged from 0.77 (FY) to 0.88 (ND50F), and the genetic advance as percentage of mean was high for yield traits (GAM = 78.67% for PY, 81.80% for GY, and 99.59% for FY). Mean grain yield across all genotypes and environments was 1,394.32 kg ha⁻¹, and the coefficient of variation ranged from 26.59% (GY) to 40.63% (FY) (Lines15-22).

Introduction — Conciseness and Redundancy

Paragraphs can be more concise. Condense duplicated ideas about drought and biotic stress

Response: We have revised the Introduction to consolidate overlapping statements on drought, low soil fertility, Striga infestation, and insect pests, which were discussed redundantly across multiple paragraphs (lines42-47).

Study Hypothesis

The objective is clearly stated, but it would help to specify the hypothesis — i.e., whether dual-purpose performance can be achieved without penalizing early maturity.

Response: An explicit hypothesis statement has been added at the end of the Introduction: among the extra-early cowpea genotypes developed at INRAN, it is possible to identify lines that combine high grain and fodder yield with stability across contrasting environments without compromising the phenological advantage of extra-early maturity—a key adaptive trait in Niger's short and erratic rainy seasons (lines75-79).

Criteria for Environment Selection

Include the criteria for selecting the six environments (e.g., agroecological differences, rainfall pattern, soil type).

Response: The three sites were selected to represent the main agro-climatic zones of southern Niger where cowpea is commercially produced. The soils across the experimental sites were predominantly sandy, although some variation in texture was observed. Maradi soil consisted of 72% sand, 11% silt, and 17% clay, while Magaria soil was more sandy, with 88% sand, 4% silt, and 8% clay. whereas Konni soils were characterized by 70% sand, 10.2-30% silt, and 15-20% clay. Soil organic carbon content was generally low across all sites, indicating poor soil fertility. Values were 0.30% at Maradi, 0.27% at Magaria, and 0.15% at Konni. Soil pH ranged from slightly acidic to near neutral, with values of 5.4 at Maradi, 5.8 at Magaria, 5.5 at Konni (lines93-103).

Balanced Data and Missing Values

Explicitly state whether data were balanced across environments, or how missing data (if any) were handled.

Response: The dataset was fully balanced across all six environments, except in Maradi (2021) no data on fodder yield was recorded.

MGIDI — Trait Weighting and Rescaling Direction

The MGIDI methodology is presented mathematically but not operationally described. Please include more detail on trait weighting and rescaling direction (which traits were desired higher or desired lower).

Response: We have expanded the MGIDI subsection in the Methods to provide full operational clarity. The ideotype was defined as a hypothetical genotype combining: maximum grain yield (GY), pod yield (PY), and fodder yield (FY)—traits for which higher values are desired (ηnj = 100, ϕnj = 0 in the rescaling formula); and minimum days to first flowering (NDF), days to 50% flowering (ND50F), and days to 50% maturity (ND50MAT)—traits for which lower values are desired (ηnj = 0, ϕnj = 100), consistent with the extra-early maturity breeding objective (lines184-189).

Rationale for Using Multiple Stability Indices

A sentence explaining why these specific stability indices were chosen (e.g., complementary strengths) would improve methodological transparency.

Response: We have added the following rationale to the Statistical Analysis subsection: These stability statistics were selected for their complementary strengths: Shukla's stability variance and Wricke's ecovalence quantify static (biological) stability by measuring variance in genotype performance across environments; the cultivar superiority index (Pi) simultaneously integrates yield level and stability by penalizing deviation from the highest-yielding genotype per environment; and the GGE biplot provides visual diagnostic capability for identifying mega-environments and comparing genotypes graphically (lines143-149).

Tables 2–3 — Biological Interpretation

Consider summarizing Tables 2–3 more narratively — focus on biological rather than statistical interpretation.

Response: The Results section accompanying Tables 2 and 3 has been revised to emphasize agronomic rather than statistical significance. For BP15 (ranked 1st on all three stability indices with a mean GY of 1,753.3 kg ha⁻¹), we now highlight its practical value as a broadly adapted genotype suitable for recommendation across all six environments tested. For BM22 (highest mean GY at 2,086.6 kg ha⁻¹ but ranked 21st for stability), indicating its suitability for deployment in high-potential, higher-rainfall environments specifically, rather than merely reporting the statistical rank contrast. Similarly, for genotypes with both low yield and poor stability (ML04, ML26, MM156, MM98), we now briefly discuss their limited breeding value and reduced priority for advancement (lines290-294).

Figure Resolution

Figures (especially GGE biplots and violin plots) need higher resolution.

Response: All figures have been regenerated at high resolution — see Response to Journal Requirement.

BM22 — High Yield but Poor Stability

Discuss briefly the discrepancy between high grain yield and poor stability observed in BM22 — what environmental or genetic factors may underlie such sensitivity?

Response: We have added a focused paragraph in the Discussion: The contrasting profile of BM22—recording the highest mean grain yield (2,086.6 kg ha⁻¹) yet ranking last (21st) for both Shukla's variance and Wricke's ecovalence—reflects specific rather than broad adaptation. BM22 was identified as a winning genotype in the high-rainfall environments of Maradi (2022) and Magaria (2022) in the Which-Won-Where biplot , but its performance declined markedly under lower-rainfall or unfertilized conditions. This pattern is consistent with a resource-responsive genotype that exploits favorable conditions efficiently but lacks the physiological buffering capacity for stable performance across heterogeneous environments. From a breeding perspective, BM22 may be of value for targeted deployment in high-rainfall zones of southeastern Niger but is not suited for broad varietal recommendation (lines449-457).

Discussion — Deeper Cause–Effect Reasoning

The discussion is sometimes descriptive; aim for deeper cause–effect reasoning (e.g., link genotype performance to rainfall variation or soil fertility where possible).

Response: The Discussion has been substantially revised to emphasize mechanistic interpretation. The contrasting fertilization regimes between 2021 (no fertilizer applied) and 2022 (NPK 15:15:15 at 6 g per plant) constituted a deliberate source of environmental variation designed to reflect the range of agronomic management conditions encountered in farmer fields in southern Niger, where fertilizer use is variable and often limited by cost. The significant year effect and GEI observed in this study are therefore partly attributable to differential genotype responses to nitrogen and phosphorus availability, in addition to the large inter-annual rainfall differences (Konni 2021: 323.5 mm vs. Magaria 2022: 741.4 mm). Genotypes that maintained relative stability under both fertilized and unfertilized conditions—notably BP15, MM65, and IT99K573-1-1 (lowest Shukla, Wi, and Pi values)—may possess greater buffering capacity against variable soil nutrient supply, a particularly valuable trait for resource-poor smallholder farming systems in Niger (lines483-493).

Remove Redundant Citations and Overlapping Explanations

Remove redundant citations or overlapping explanations of the same concepts (e.g., stability interpretation repeated from Results).

Response: We have reviewed the entire manuscript and removed passages in the Discussion that unnecessarily restated stability definitions or interpretations already provided in the Methods or Results.

Practical Breeding Implications

Include more practical breeding implications: how these genotypes might integrate into current INRAN or regional breeding pipelines

Response: We have added a dedicated paragraph in the Discussion on breeding pipeline integration: These results have direct implications for INRAN's cowpea breeding pipeline. The ten selected genotypes are candidates for submission to INRAN's national performance, the formal prerequisite for variety release in Niger. Cross-border multi-environment evaluation in collaboration with IITA-West Africa and national programs in Mali and Burkina Faso—where similar semi-arid production conditions prevail—would further validate their performance across a broader range of agroecological zones. Participatory variety selection (PVS) trials with smallholder farmers in Niger are recommended to validate farmer acceptance of these dual-purpose lines before formal recommendation (lines494-501).

Conclusion — Implications Over Results

Avoid restating results verbatim in the Conclusion. Focus instead on implications (climate resilience, early adoption, need for participatory trials).

Response: The Conclusion section has been rewritten: this study demonstrates that extra-early maturity and dual-purpose productivity are compatible traits in cowpea a finding of direct relevance for climate adaptation in the Sahel, where shortening rainy seasons increasingly favor short-duration crops. The ten genotypes identified through combined stability analysis and the MGIDI index (BM22, BP02, BP15, MM142, 65B5080, BP18, BM21, BM13, MM141, and BM12) are promising candidates for further evaluation. However, given that trials were conducted over two growing seasons across six site-year combinations, multi-season and multi-location validation across additional agroecological zones is required before formal variety release recommendations can be made. Future research should prioritize participatory variety selection with smallholder farmers in the target regions to ensure that performance advantages translate into adoption outcomes, and to validate farmer preferences for grain quality, fodder quantity, and compatibility with local agronomic practices. These efforts, combined with cross-border collaboration, will be essential for maximizing the impact of these genotypes on food and feed security in Niger and the wider Sahel (lines503-515).

III. REVIEWER #2 COMMENT

Criteria for Classifying Genotypes as High-Yielding and Stable

The criteria used to classify genotypes as both high yielding and stable should be explained more clearly. A summary table integrating yield performance and all stability metrics would improve interpretation.

Response: A paragraph has been added to the Results: a genotype was classified as high-yielding if its mean grain yield exceeded the grand mean (1,394.32 kg ha⁻¹), and as stable if it ranked in the top third (≤ 7 out of 21) on at least two of the three stability indices simultaneously (lines279-281). The existing Table 2 has been already to present mean GY alongside all three stability ranks in a single integrated table.

Environmental Characterization

The environmental characteristics of the six testing sites are not sufficiently described. Additional information on rainfall, soil properties, and climatic conditions would help readers understand sources of GEI.

Response: We have added a dedicated environmental characterization: The three sites were selected to represent the main agro-climatic zones of southern Niger where cowpea is commercially produced. The soils across the experimental sites were predominantly sandy, although some variation in texture was observed. Maradi soil consisted of 72% sand, 11% silt, and 17% clay, while Magaria soil was more sandy, with 88% sand, 4% silt, and 8% clay. whereas Konni soils were characterized by 70% sand, 10.2-30% silt, and 15-20% clay. Soil organic carbon content was generally low across all sites, indicating poor soil fertility. Values were 0.30% at Maradi, 0.27% at Magaria, and 0.15% at Konni. Soil pH ranged from slightly acidic to near neutral, with values of 5.4 at Maradi, 5.8 at Magaria, 5.5 at Konni (lines93-103).

Limited Scope — Two Year

Attachment Submitted filename: Response to Reviewers.pdf

# Editor report: Decision Letter 1

**DOI:** 10.1371/journal.pone.0354617.r004

**Authors:** Muhammad Amjad Ali

5 Jul 2026

<div>PONE-D-25-68979R1

Stability of grain and fodder yields in extra-early cowpea genotypes in Southeastern Niger

PLOS One

Dear Dr. Moumouni,

Thank you for submitting your manuscript to PLOS ONE. After careful consideration, we feel that it has merit but does not fully meet PLOS ONE’s publication criteria as it currently stands. Therefore, we invite you to submit a revised version of the manuscript that addresses the points raised during the review process.

Please submit your revised manuscript by Aug 19 2026 11:59PM. If you will need more time than this to complete your revisions, please reply to this message or contact the journal office at plosone@plos.org. When you're ready to submit your revision, log on to https://www.editorialmanager.com/pone/ and select the 'Submissions Needing Revision' folder to locate your manuscript file.

Please include the following items when submitting your revised manuscript:

- A letter that responds to each point raised by the academic editor and reviewer(s). You should upload this letter as a separate file labeled 'Response to Reviewers'.
- A marked-up copy of your manuscript that highlights changes made to the original version. You should upload this as a separate file labeled 'Revised Manuscript with Track Changes'.
- An unmarked version of your revised paper without tracked changes. You should upload this as a separate file labeled 'Manuscript'.

If you would like to make changes to your financial disclosure, please include your updated statement in your cover letter. Guidelines for resubmitting your figure files are available below the reviewer comments at the end of this letter.

If applicable, we recommend that you deposit your laboratory protocols in protocols.io to enhance the reproducibility of your results. Protocols.io assigns your protocol its own identifier (DOI) so that it can be cited independently in the future. For instructions see: https://journals.plos.org/plosone/s/submission-guidelines#loc-laboratory-protocols. Additionally, PLOS ONE offers an option for publishing peer-reviewed Lab Protocol articles, which describe protocols hosted on protocols.io. Read more information on sharing protocols at https://plos.org/protocols?utm_medium=editorial-email&utm_source=authorletters&utm_campaign=protocols.

As the corresponding author, your ORCID iD is verified in the submission system and will appear in the published article. PLOS supports the use of ORCID, and we encourage all coauthors to register for an ORCID iD and use it as well. Please encourage your coauthors to verify their ORCID iD within the submission system before final acceptance, as unverified ORCID iDs will not appear in the published article. *Only*  the individual author can complete the verification step; PLOS staff *cannot*  verify ORCID iDs on behalf of authors.

We look forward to receiving your revised manuscript.

Kind regards,

Muhammad Amjad Ali, PhD

Academic Editor

PLOS One

Journal Requirements:

If the reviewer comments include a recommendation to cite specific previously published works, please review and evaluate these publications to determine whether they are relevant and should be cited. There is no requirement to cite these works unless the editor has indicated otherwise.

Please review your reference list to ensure that it is complete and correct. If you have cited papers that have been retracted, please include the rationale for doing so in the manuscript text, or remove these references and replace them with relevant current references. Any changes to the reference list should be mentioned in the rebuttal letter that accompanies your revised manuscript. If you need to cite a retracted article, indicate the article’s retracted status in the References list and also include a citation and full reference for the retraction notice.

Additional Editor Comments:

Although the authors have significantly improved the manuscript, the relevant and recent citations are still missing, I would suggest to cite the following relevant citations.

Munir, R., M. Kashif, M.Z. Iqbal and G. Farid. 2024. Stability Analysis of Genotype × Environment Interaction for Wheat Genotypes Through Bayesian and Frequentist Approaches. Pak. J. Agri. Sci., Vol. 61(4), 1013-1021

[Note: HTML markup is below. Please do not edit.]

[NOTE: If reviewer comments were submitted as an attachment file, they will be attached to this email and accessible via the submission site. Please log into your account, locate the manuscript record, and check for the action link "View Attachments". If this link does not appear, there are no attachment files.]

To ensure your figures meet our technical requirements, please review our figure guidelines: https://journals.plos.org/plosone/s/figures

You may also use PLOS’s free figure tool, NAAS, to help you prepare publication quality figures: https://journals.plos.org/plosone/s/figures#loc-tools-for-figure-preparation.

NAAS will assess whether your figures meet our technical requirements by comparing each figure against our figure specifications.

# Author comment: Author response to Decision Letter 2

**DOI:** 10.1371/journal.pone.0354617.r005

8 Jul 2026

Dear Editors-in-Chief,

I am writing to submit the revised version of the manuscript titled " Stability of grain and fodder yields in extra-early cowpea genotypes in southeastern Niger " following Editor’s comments and suggestions.

We really appreciate the editor for the opportunity to further revise our manuscript. We have carefully addressed the additional comment raised. The changes have been incorporated into the revised manuscript and are highlighted in the tracked-changes version.

Journal Requirements

Please review your reference list to ensure that it is complete and correct

Response:

We have reviewed the full reference list and confirm that it is complete and correct. None of the cited references have been retracted. One new reference (Munir et al., 2024, entry #60) has been added in response to the Academic Editor's comment; no other changes were made to the reference list.

Academic Editor Comment

Although the authors have significantly improved the manuscript, the relevant and recent citations are still missing, I would suggest to cite the following relevant citations.

Munir, R., M. Kashif, M.Z. Iqbal and G. Farid. 2024. Stability Analysis of Genotype × Environment Interaction for Wheat Genotypes Through Bayesian and Frequentist Approaches. Pak. J. Agri. Sci., Vol. 61(4), 1013-1021.

Response: We thank the Academic Editor for this suggestion. We have reviewed Munir et al. (2024) and agree that it is relevant, as it also reports highly significant genotype, environment, and genotype × environment interaction effects from analysis of variance in multi-environment trials and compares Bayesian and frequentist approaches for stability analysis. We have cited this reference in the Discussion section, where we compare our GEI findings with reports from other cropping systems, to reinforce that significant G×E effects are a broadly observed across crops and not specific to cowpea (lines462-465).

The full reference has been added to the reference list as entry #60: Munir R, Kashif M, Iqbal MZ, Farid G. 2024. Stability Analysis of Genotype × Environment Interaction for Wheat Genotypes Through Bayesian and Frequentist Approaches. Pak J Agri Sci. 61:1013–1021. https://doi.org/10.21162/PAKJAS/24.276

We believe the revised manuscript is now substantially improved and we look forward to the Editor's further consideration.

Sincerely,

Abdoul Moumouni Iro Sodo

abdoulmoumouniirosodo@gmail.com

Attachment Submitted filename: Response_to_Reviewers_auresp_2.pdf

# Editor report: Decision Letter 2

**DOI:** 10.1371/journal.pone.0354617.r006

**Authors:** Muhammad Amjad Ali

9 Jul 2026

Stability of grain and fodder yields in extra-early cowpea genotypes in Southeastern Niger

PONE-D-25-68979R2

Dear Dr. Moumouni,

We’re pleased to inform you that your manuscript has been judged scientifically suitable for publication and will be formally accepted for publication once it meets all outstanding technical requirements.

Within one week, you’ll receive an e-mail detailing the required amendments. When these have been addressed, you’ll receive a formal acceptance letter and your manuscript will be scheduled for publication.

An invoice will be generated when your article is formally accepted. Please note, if your institution has a publishing partnership with PLOS and your article meets the relevant criteria, all or part of your publication costs will be covered. Please make sure your user information is up-to-date by logging into Editorial Manager at Editorial Manager® and clicking the ‘Update My Information' link at the top of the page. For questions related to billing, please contact billing support.

If your institution or institutions have a press office, please notify them about your upcoming paper to help maximize its impact. If they’ll be preparing press materials, please inform our press team as soon as possible -- no later than 48 hours after receiving the formal acceptance. Your manuscript will remain under strict press embargo until 2 pm Eastern Time on the date of publication. For more information, please contact onepress@plos.org.

Kind regards,

Muhammad Amjad Ali, PhD

Academic Editor

PLOS One

Additional Editor Comments (optional):

Reviewers' comments:

# Editor report: Acceptance letter

**DOI:** 10.1371/journal.pone.0354617.r007

**Authors:** Muhammad Amjad Ali

PONE-D-25-68979R2

PLOS One

Dear Dr. Iro Sodo,

I'm pleased to inform you that your manuscript has been deemed suitable for publication in PLOS One. Congratulations! Your manuscript is now being handed over to our production team.

At this stage, our production department will prepare your paper for publication. This includes ensuring the following:

* All references, tables, and figures are properly cited

* All relevant supporting information is included in the manuscript submission,

* There are no issues that prevent the paper from being properly typeset

You will receive further instructions from the production team, including instructions on how to review your proof when it is ready. Please keep in mind that we are working through a large volume of accepted articles, so please give us a few days to review your paper and let you know the next and final steps.

Lastly, if your institution or institutions have a press office, please let them know about your upcoming paper now to help maximize its impact. If they'll be preparing press materials, please inform our press team within the next 48 hours. Your manuscript will remain under strict press embargo until 2 pm Eastern Time on the date of publication. For more information, please contact onepress@plos.org.

You will receive an invoice from PLOS for your publication fee after your manuscript has reached the completed accept phase. If you receive an email requesting payment before acceptance or for any other service, this may be a phishing scheme. Learn how to identify phishing emails and protect your accounts at https://explore.plos.org/phishing.

If we can help with anything else, please email us at customercare@plos.org.

Thank you for submitting your work to PLOS ONE and supporting open access.

Kind regards,

PLOS ONE Editorial Office Staff

on behalf of

Dr. Muhammad Amjad Ali

Academic Editor

PLOS One
